# Ultra-processed food intake and risk of digestive system cancers: a systematic review and dose–response meta-analysis

**DOI:** 10.3389/fnut.2026.1901660

**Published:** 2026-07-15

**Authors:** Yanjie Jiang, Shipeng Zhang, Hanyu Wang, Xingyi He, Zhihui Jin, Rui Fu, Qinwei Fu, Wenshan Li, Xiaoyu Zhu, Zhuohong Li, Xin Sun, Yan Lu

**Affiliations:** 1 Nanjing Hospital of Chinese Medicine Affiliated to Nanjing University of Chinese Medicine, Nanjing, China; 2 Hospital of Chengdu University of Traditional Chinese Medicine, Chengdu University of Traditional Chinese Medicine, Chengdu, China; 3 The First Clinical Medical College, Guangzhou University of Chinese Medicine, Guangzhou, China; 4 Department of Health Research Methods, Evidence, and Impact (HEI), McMaster University, Hamilton, ON, Canada

**Keywords:** digestive system cancers, dose–response meta-analysis, NOVA classification, systematic review, ultra-processed foods (UPF)

## Abstract

**Background:**

Ultra-processed food (UPF) intake has been associated with digestive system cancer risk, but evidence remains scattered across cancer sites, study designs, and exposure metrics. This systematic review and meta-analysis evaluated associations between UPF intake and digestive system cancer risk overall and by cancer site. We further examined UPF subcategories and dose–response relationships.

**Methods:**

We systematically searched PubMed, Embase, Web of Science, and the Cochrane Library for English-language cohort and case–control studies published from database inception to May 1, 2026. Hazard ratios (HRs) from cohort studies and odds ratios (ORs) from case–control studies were pooled separately with their corresponding 95% confidence intervals (CIs) using random-effects models. Additional analyses were conducted to investigate site-specific digestive system cancers, UPF subcategories, and dose–response relationships, alongside subgroup, sensitivity, meta-regression, and publication bias assessments. The study protocol was registered in PROSPERO (CRD420261404299).

**Results:**

A total of nine prospective cohort studies and five case–control studies were included. The main pooled estimates showed a 12% higher risk in cohort studies (HR = 1.12, 95% CI: 1.05–1.20; *I*^2^ = 37.6%) and 42% higher odds in case–control studies (OR = 1.42, 95% CI: 1.25–1.60; *I*^2^ = 26.7%). Site-specific analyses showed relatively consistent positive associations for colorectal and colon cancer outcomes. Among UPF subcategories, ultra-processed meat/protein products were associated with higher digestive system cancer risk (HR = 1.33, 95% CI: 1.15–1.53). In dose–response analyses using UPF weight share as the primary exposure metric, each 10-percentage-point increase in UPF intake was associated with a 6% higher risk (HR = 1.06, 95% CI: 1.01–1.10), with evidence of non-linearity, although no reliable threshold could be identified. Supplementary analyses based on g/day showed a consistent direction of association. Sensitivity analyses supported the stability of the main findings, and no clear evidence of publication bias was observed.

**Conclusion:**

Higher UPF intake was associated with increased digestive system cancer risk, with relatively consistent evidence for colorectal and colon cancers. Ultra-processed meat/protein products may be a relevant contributing subcategory, and dose–response analyses suggested an exposure–response gradient. Because evidence came mainly from observational studies and dose–response data were limited, findings require confirmation in high-quality prospective studies.

**Systematic Review Registration:**

https://www.crd.york.ac.uk/PROSPERO/view/CRD420261404299, identifier PROSPERO (CRD420261404299).

## Graphical abstract

**Figure:**
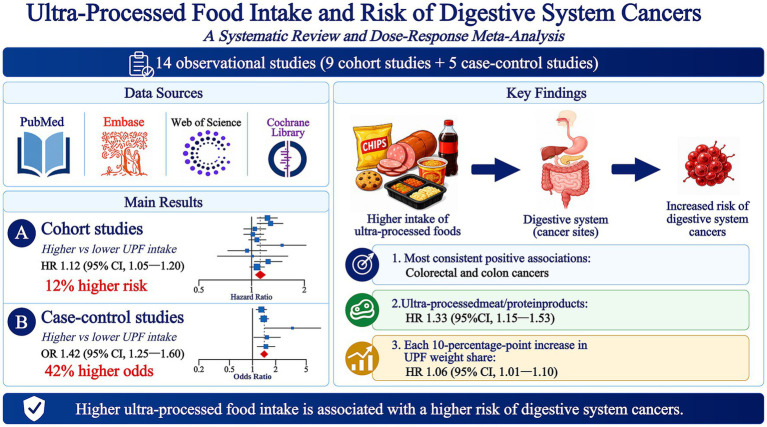
Summary of the main findings of this systematic review and dose–response meta-analysis.

## Introduction

Digestive system cancers constitute a major component of the global cancer burden, accounting for 39.29% of cancer-related deaths worldwide. Colorectal, gastric, liver, oesophageal, pancreatic, and gallbladder cancers are major contributors, with substantial geographic variation in incidence and mortality (1, 2). Dietary exposures are key modifiable factors in the primary prevention of digestive system cancers (3). Previous epidemiological studies have largely assessed diet in terms of individual nutrients, food groups, or overall dietary patterns, showing that higher-quality diets, better carbohydrate quality, and greater plant-based food intake may be associated with lower risks of some digestive system cancers (3–6). However, amid global food system transformations and the growing availability and consumption of industrially processed foods, UPF intake has risen substantially, increasingly displacing minimally processed foods such as whole grains, legumes, fresh fruits, and vegetables (7, 8). These shifts indicate that nutrient- or food group-based approaches may not fully capture diet-related cancer risk, and that food processing level represents an additional dimension for characterising dietary exposures in chronic disease research (9, 10).

Monteiro et al. (11, 12) developed the NOVA food classification system, which classifies foods according to the extent and purpose of industrial processing and provides a structured basis for defining UPF and assessing diets according to food processing level. Higher UPF intake is often characterised by poorer dietary quality, excess energy intake, and greater exposure to additives and processing-related contaminants. It may also contribute to digestive system cancer risk through obesity-related metabolic dysregulation, gut microbiota alterations, impaired mucosal barrier function, and chronic inflammation (13–15). Prospective studies suggest that UPF intake may be associated with higher risks of overall digestive system cancer and several site-specific digestive system cancers (16, 17). However, evidence remains scattered across studies of overall cancer, individual cancer sites, and cancer mortality, with limited synthesis across the full spectrum of digestive system cancers or dose–response relationships (9). Given the heterogeneity of digestive system cancers by anatomical site, aetiology, and dietary sensitivity, we pooled cohort and case–control studies separately. We evaluated associations between UPF intake and digestive system cancer risk overall and by cancer site, and further examined UPF subcategories and dose–response relationships. The overall study design and principal findings are summarised in the Graphical Abstract.

## Methods

### Registration of review protocol

This study was conducted and reported in accordance with the Preferred Reporting Items for Systematic Reviews and Meta-Analyses (PRISMA) statement (18). The protocol was registered in the International Prospective Register of Systematic Reviews (PROSPERO; CRD420261404299). As this study used previously published aggregate data and did not involve individual-level primary data collection, ethical approval was not required.

### Search strategy

We systematically searched PubMed, Embase, Web of Science, and the Cochrane Library from database inception to May 1, 2026. The search was restricted to English-language publications. The search strategy combined three core concepts: UPF exposure, cancer-related terms, and controlled vocabulary and free-text terms for digestive system cancers. Key terms included “ultra-processed foods,” “UPF,” “NOVA classification,” “food processing,” and “processed food” for exposure; “neoplasm,” “cancer,” “tumor,” “malignancy,” “carcinoma,” and “adenocarcinoma” for cancer outcomes; and digestive or gastrointestinal neoplasm terms and site-specific terms for colorectal, colon, rectal, gastric, pancreatic, oesophageal, hepatic, biliary, gallbladder, and ampullary cancers. We also manually screened the reference lists of relevant articles to identify additional eligible studies. The complete search strategies are provided in the Supplementary material.

### Eligibility criteria

According to previous recommendations (19), the review question was defined using the PECO-S framework (20). P-Population: adult populations without restrictions on specific disease status; E-Exposure: intake of ultra-processed foods or ultra-processed diets, preferably defined according to NOVA group 4 of the NOVA food classification system; C-Comparison: populations with lower UPF intake, including the lowest intake category or a study-defined low-intake reference group; O-Outcome: incident overall and site-specific digestive system cancers, including colorectal, colon and rectal, oesophageal and histological subtypes, gastric, liver and hepatocellular, biliary tract, gallbladder, and pancreatic cancers; and S-Study design: cohort studies and case–control studies. Cross-sectional studies were excluded because they cannot establish temporality and are more susceptible to reverse causation.

The following exclusion criteria were applied: (1) duplicate publications or repeated reports from the same cohort; (2) studies for which the full text or key data were unavailable; (3) studies with irrelevant exposures, such as those evaluating processed foods in general, individual foods, or dietary patterns without explicitly assessing UPF intake; (4) studies with irrelevant outcomes, such as precancerous lesions, colorectal adenomas, polyps, or cancers outside the digestive system; (5) studies with irrelevant designs, including intervention studies, cross-sectional studies, ecological studies, study protocols, reviews, commentaries, or case reports. When different disease outcomes were reported from the same cohort, they were treated as separate outcome-specific cohorts (Supplementary Table S1).

### Study selection

Study selection was conducted in two sequential stages. First, two investigators (YJ and SZ) imported all retrieved records into EndNote X9 and removed duplicates. They then independently screened titles and abstracts against the predefined eligibility criteria to identify potentially eligible studies. The same two investigators subsequently reviewed the full texts and determined the studies eligible for the systematic review and quantitative synthesis. Any discrepancies were resolved by discussion with a third investigator (HW) until consensus was reached.

### Data extraction

Data extraction was performed independently by two investigators (YJ and SZ), and discrepancies were resolved through discussion with a third investigator (HW). When information was unclear or key data were missing, we contacted the corresponding authors of the original studies. We extracted the following data: first author, publication year, geographic region, study design, cohort source, study period, mean follow-up duration, participant characteristics (proportion of women, baseline age, and BMI), total energy intake, UPF assessment tool, UPF exposure unit, mean UPF intake, digestive system cancer outcome, number of cases, total sample size, effect estimates with 95% CIs, adjustment status, and adjusted covariates.

For cohort studies, multivariable-adjusted HRs with 95% CIs were preferentially extracted; for case–control studies, multivariable-adjusted ORs with 95% CIs were preferentially extracted. When multiple adjustment models were reported, the model with the most comprehensive confounder adjustment was selected. For results reported in non-overlapping subgroups within the same study, such as sex-specific estimates, a fixed-effect model was used to combine them into a single estimate to avoid double-counting participants.

### Data synthesis and analysis

The primary meta-analysis compared the highest versus lowest UPF intake categories. Because cohort and case–control studies differ in design, bias structure, and interpretation of effect estimates, they were synthesised separately: HRs for cohort studies and ORs for case–control studies. DerSimonian–Laird random-effects models were used for the primary pooled estimates to evaluate associations between UPF intake and overall and site-specific digestive system cancer risk (21). Continuous effect estimates for UPF exposure reported in the original studies were also extracted. Because exposure units varied, including servings/day, g/day, percentage of energy intake, and UPF weight share, quantitative synthesis was restricted to UPF weight share, for which study numbers were sufficient and definitions were relatively consistent; estimates were standardised per 10-percentage-point increase in UPF weight share. Between-study heterogeneity was assessed using Cochran’s *Q* test, *τ*^2^ for between-study variance, and *I*^2^ for the degree of heterogeneity (22, 23). Site-specific analyses were conducted separately by study design and pooled only when at least two studies reported the same cancer subtype; eligible subtypes are listed in Supplementary Table S2. Subgroup analyses were stratified by sex, geographic region, mean follow-up duration, mean age, mean BMI, mean total energy intake, and dietary assessment method in cohort studies, and by geographic region, mean total energy intake, and control source in case–control studies. Differences between subgroups were assessed using between-group *χ*^2^ tests. Meta-regression analyses were conducted to explore whether study-level characteristics, UPF exposure units, main-model covariate adjustments, and the number of adjusted covariates contributed to variation in effect estimates. For cohort studies, we also summarised associations between UPF subcategory intake and digestive system cancer risk; quantitative synthesis was not performed for case–control studies because UPF subcategory definitions and reporting methods were inconsistent. Leave-one-out sensitivity analyses were conducted for the primary pooled estimates to assess the influence of individual studies. Publication bias was assessed using funnel plots and Egger’s regression test (24); when indicated, the trim-and-fill method was applied to evaluate its potential influence (25). All statistical tests were two-sided; *p* < 0.05 was considered statistically significant.

In dose–response analyses, we evaluated linear and non-linear associations between UPF intake and digestive system cancer risk. For studies reporting at least three UPF exposure levels with corresponding risk estimates, study-specific dose–response trends were estimated using the Greenland and Longnecker generalised least-squares trend method and pooled with random-effects models (26, 27). Linear dose–response results were expressed as HRs and 95% CIs per 10-percentage-point increase in UPF weight share and per 100 g/day increase in absolute UPF intake. Non-linear dose–response associations were modelled using restricted cubic splines, with knots at the 10th, 50th, and 90th percentiles of the exposure distribution, as recommended by Harrell. This approach incorporates study-specific trends into a single model to estimate the overall dose–response association and assess potential non-linearity (28). Non-linearity was assessed by testing whether the coefficient of the second spline term differed from zero (29). A similar approach has been applied in previous dose–response meta-analyses (30).

### Evaluation of study quality

The methodological quality of included studies was assessed using the Newcastle–Ottawa Scale (NOS) (31). Cohort and case–control studies were evaluated using the corresponding NOS versions. The NOS comprises three domains: selection, comparability, and outcome or exposure assessment, with total scores ranging from 0 to 9. Scores of 0–3, 4–6, and 7–9 were classified as low, moderate, and high methodological quality, respectively. Quality assessment was performed independently by two investigators, and disagreements were resolved through discussion with a third investigator.

In addition, the certainty of evidence for each outcome was assessed using the Grading of Recommendations Assessment, Development and Evaluation (GRADE) approach and classified as high, moderate, low, or very low (32). Cohen’s kappa statistic was used to assess inter-investigator agreement in study selection, data extraction, and quality assessment. Kappa values were interpreted according to Landis and Koch criteria (33): <0, poor agreement; 0.00–0.20, slight agreement; 0.21–0.40, fair agreement; 0.41–0.60, moderate agreement; 0.61–0.80, substantial agreement; and 0.81–1.00, almost perfect agreement.

### Software, data, and code availability

Meta-analyses were performed using Stata version 16.0. Dose–response analyses were conducted using R software version 4.4.2 with the dosresmeta and mvmeta packages, and visualisations were generated with ggplot2.

## Results

### Literature search

**Figure 1:**
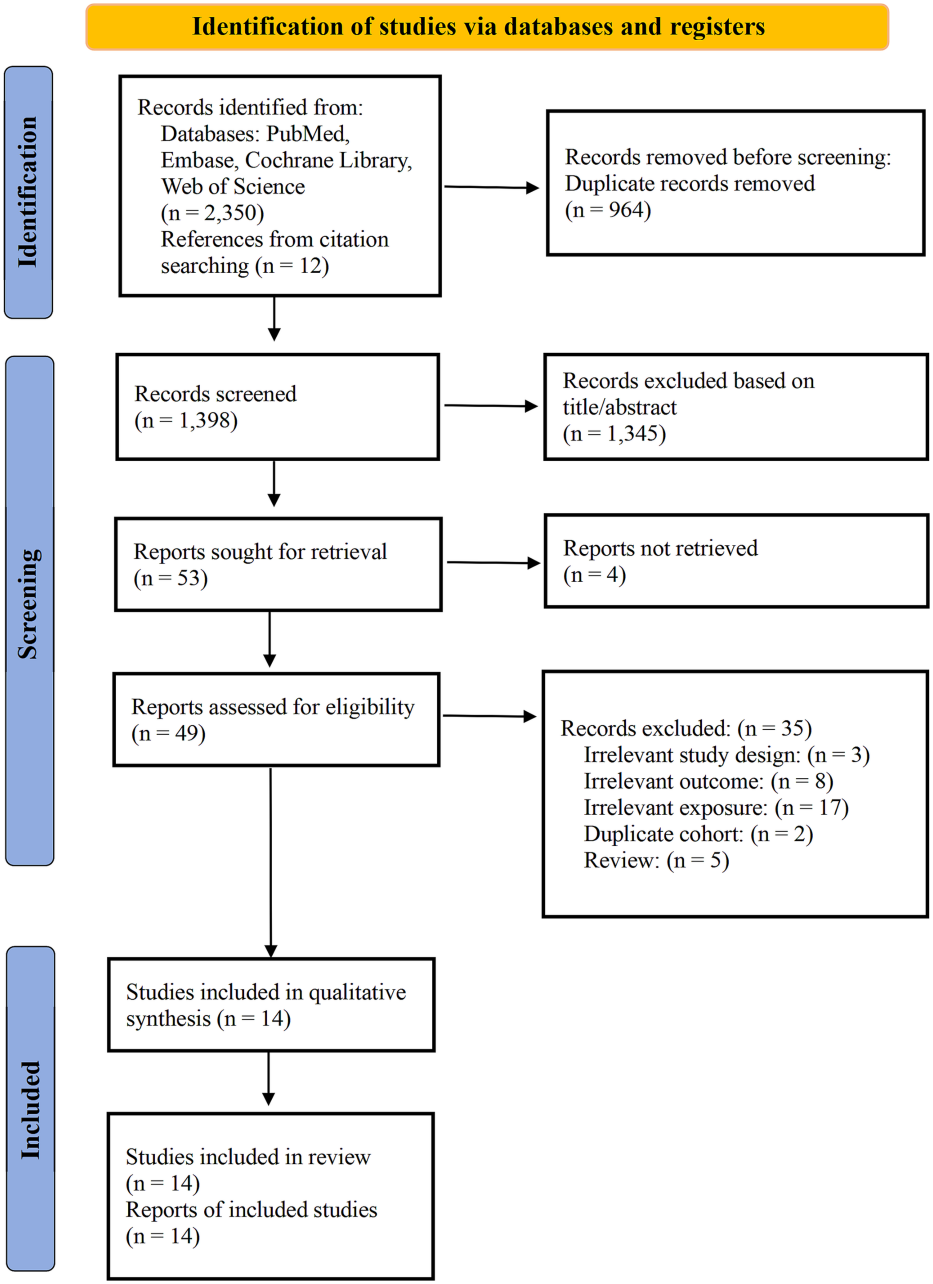
PRISMA flowchart of study inclusion and exclusion.

Figure 1 shows the PRISMA flow diagram for study selection. A total of 2,362 potentially relevant records were identified through database and citation searches. After removal of 964 duplicates, titles and abstracts were screened, and 53 articles were retained for full-text assessment. After full-text review, 35 articles were excluded for the reasons listed below; details of excluded studies are provided in Supplementary Table S3. Finally, 14 studies reporting associations between UPF intake and digestive system cancer risk were included in this meta-analysis. The kappa value for study selection between the two reviewers was 0.815, indicating almost perfect agreement.

### Study characteristics

Table 1 summarises the main characteristics of the nine prospective cohort studies (16, 17, 34–40) and five case–control studies (41–45) included in this meta-analysis. The included studies were published from 2018 to 2026. The nine cohort studies, which included NutriNet-Santé, NHS/NHS II, HPFS, EPIC, UK Biobank, PLCO, SCCS, and NOWAC, were conducted mainly in France, the United States, the United Kingdom, Norway, and in multinational European cohorts. At the study level, cohort sample sizes ranged from 73,119 to 450,111 participants, and mean or median follow-up ranged from 5.0 to 26.6 years. Mean age ranged from 42.8 to 65.6 years, BMI from 23.8 to 30.4 kg/m^2^, and mean dietary energy intake from 1682.0 to 2548.9 kcal/day. UPF intake was assessed using repeated 24-h dietary records or recalls, semi-quantitative food frequency questionnaires, dietary questionnaires specific to individual countries or study centres, and diet history questionnaires; exposure units included servings/day, g/day, UPF weight share, and each 10-percentage-point increase in UPF weight share. Most cohort studies adjusted for demographic, lifestyle, dietary, and clinical confounders in multivariable models.

**Table 1:** Characteristics of included studies.

Author, year	Study design	Geographic region	Data source	Study period	Follow-up duration, years	Study population	Female proportion, %	Baseline age, years	BMI, kg/m^2^	Energy intake, kcal/day	UPF assessment and exposure metric	Outcome	Sample size	Main covariates adjusted for
Fiolet et al. (34)	Cohort	France	NutriNet-Sante prospective cohort	2009–2017	5.0	Healthy adults	78.3	42.8	23.8	1879	Repeated web-based 24-h dietary records; NOVA; %g/day	Colorectal cancer	104,980	Age, sex, energy intake, number of dietary records, smoking, education, physical activity, height, BMI, alcohol, family history of cancer, and dietary factors
Wang et al. (35)	Cohort	United States	HPFS	1986–2014	23.2	Healthy adults	0	54.0	25.3	1957.0	Validated semiquantitative FFQ; NOVA; servings/day	Colorectal, proximal colon, distal colon, and rectal cancer	46,341	Age, calendar year, race, family history of cancer, endoscopy, alcohol intake, physical activity, smoking, energy intake, aspirin use
Wang et al. (35)	Cohort	United States	NHS, NHS II,	1986–2015	26.6	Healthy adults	100	52.6	24.9	1750.8	Validated semiquantitative FFQ; NOVA; servings/day	Colorectal, proximal colon, distal colon, and rectal cancer	159,907	Age, calendar year, race, family history of cancer, endoscopy, alcohol intake, physical activity, smoking, energy intake, aspirin use, menopausal status, hormone use
Zhong et al. (36)	Cohort	United States	PLCO Cancer Screening Trial	1993–2009	8.9	Adults aged 55–74 years	52.5	65.6	27.2	1713.1	Diet History Questionnaire; NOVA; servings/day	Pancreatic cancer	98,265	Age, sex, race/ethnicity, smoking, alcohol, BMI, aspirin use, diabetes, family history of pancreatic cancer, and energy intake
Chang et al. (16)	Cohort	United Kingdom	UK Biobank	2007–2021	9.8	Adults aged 40–69 years	54.6	58.0	26.4	2044.0	Web-based 24-h dietary recall; NOVA; %g/day	Gastrointestinal cancer, oesophagus cancer, oesophageal adenocarcinoma, oesophageal squamous cell carcinoma, stomach cancer, stomach cardia cancer, stomach non-cardia cancer, small intestine cancer, colorectal cancer, colon cancer, rectum cancer, anal cancer, hepatobiliary tract cancer, liver cancer, hepatocellular carcinoma, intrahepatic bile duct cancer, and pancreatic cancer	197,426	Age, ethnicity, smoking, physical activity, income, education, alcohol, BMI, energy intake, sex, height, family history of cancer, deprivation index, and region
Kliemann et al. 2023 (17)	Cohort	Europe	EPIC cohort	1991–2013	14.1	Adults without cancer at baseline	70.8	51.1	25.3	2076.0	Dietary questionnaires specific to individual countries or study centres/FFQs; NOVA; g/day and %g/day	Colorectal, proximal colon, distal colon, rectal cancer, oesophageal adenocarcinoma, oesophageal squamous cell carcinoma, gastric cardia cancer, non-cardia gastric cancer, hepatocellular carcinoma, gallbladder cancer, and pancreatic cancer	450,111	Age, centre, sex, smoking, education, physical activity, height, diabetes, BMI, Mediterranean diet, alcohol, energy intake, fat, sodium, and carbohydrate intake
Zhao et al. (37)	Cohort	United States	SCCS	2002–2019	13.9	Adults aged 40–79 years	59.8	52.1	30.4	2548.9	Validated semiquantitative FFQ; NOVA; % food weight (%g/day)	Liver cancer	73,119	Age, sex, race, income, education, smoking, alcohol, physical activity, aspirin/statin use, family history, hepatitis B/C, diabetes, energy intake, and BMI
Zhao et al. (38)	Cohort	United Kingdom	UK Biobank	2006–2020	8.9	Adults aged 40–69 years	54.3	56.0	26.9	2044.4	24-h dietary recalls; NOVA; energy-adjusted g/day	Liver cancer	173,889	Age, sex, ethnicity, Townsend deprivation index, smoking, alcohol, physical activity, BMI, aspirin use, diabetes, and energy intake
Mols et al. (39)	Cohort	Norway	NOWAC	1991–2018	17.4	Women aged 30–70 years	100	51.3	24.7	1682.0	Validated semiquantitative FFQ; NOVA; g/day	Colorectal, colon, proximal colon, distal colon, and rectal cancer	77,100	Age, education, smoking, height, menopausal hormone therapy, physical activity, and energy intake
Al Nahas et al. (40)	Cohort	Europe	EPIC cohort	1992–2013	14.1	Adults without cancer at baseline	70.8	51.1	25.3	NA	Dietary questionnaires specific to individual countries or study centres/FFQs; NOVA; %g/day; baseline g/day	Colorectal, colon, proximal colon, distal colon, and rectal cancer	450,111	Age, sex, centre, smoking, education, physical activity, height, BMI, Mediterranean diet, alcohol, energy intake, calcium, and fibre intake
Romaguera et al. (41)	Case–control	Spain	MCC-Spain population-based case–control study	2008–2013	NA	Adults aged 20–85 years	49.9	62.9	26.9	1932.3	Validated 140-item semiquantitative FFQ; NOVA; % of total daily food weight	Colorectal, colon, and rectal cancer	5,241	Sex, age, study area, education, BMI, physical activity, smoking, NSAID use, family history of CRC, energy intake, and ethanol intake
El Kinany et al. (42)	Case–control	Morocco	Nationally representative multicentre case–control study	NA	NA	Adults	50.7	55.5	25.2	3247.4	Validated semiquantitative FFQ; NOVA; g/day	Colorectal, colon, and rectal cancer	2,906	Age, education, family history of CRC, smoking, physical activity, BMI, energy intake, and other NOVA food groups
Jafari et al. (43)	Case–control	Iran	Hospital-based matched case–control study	2008–2010	NA	Adults aged 40–75 years	NA	NA	26.8	2257.6	125-item semiquantitative FFQ; NOVA; study-defined categories	Colorectal cancer	213	BMI, income, smoking, occupation, education, and exercise
Du et al. (44)	Case–control	United States	NHS and HPFS nested case–control study	1976–2001	12.5	Adults	60.2	61.5	NA	1929.0	Validated FFQ; NOVA; servings/day	Colorectal cancer	1,372	Age at blood draw, sex, race, fasting status, energy intake, alcohol, physical activity, smoking, menopausal factors, aspirin use, BMI, AHEI-2010, fruit and vegetable intake
Torres-Collado et al. (45)	Case–control	Spain	PANESOES hospital-based multi-case–control study	1995–1999	NA	Spanish-speaking adults aged 30–80 years	32.0	64.5	NA	1959.8	Validated semiquantitative FFQ; NOVA; g/day	Gastrointestinal cancer, oesophageal cancer, stomach cancer, and pancreatic cancer	1,218	Age, sex, province, education, smoking, and NOVA groups 1–3

Studies are ordered by study design, with cohort studies listed first followed by case–control studies; within each study design, studies are arranged chronologically by publication year. Wang et al. 2022a and 2022b represent men and women from the same study, respectively, whereas Zhao et al. (37, 38) represent two distinct cohort studies. The UPF assessment and exposure metric column is presented as dietary assessment tool; UPF classification; metric used for effect estimation. %g/day indicates the percentage contribution of UPF to total food intake by weight.

AHEI, Alternative Healthy Eating Index; BMI, body mass index; CRC, colorectal cancer; EPIC, European Prospective Investigation into Cancer and Nutrition; FFQ, food frequency questionnaire; HPFS, Health Professionals Follow-up Study; NA, not applicable or not reported; NHS, Nurses’ Health Study; NOWAC, Norwegian Women and Cancer Study; PLCO, Prostate, Lung, Colorectal and Ovarian Cancer Screening Trial; SCCS, Southern Community Cohort Study; UPF, ultra-processed food.

The five case–control studies were conducted in Spain, Morocco, Iran, and the United States, with sample sizes ranging from 213 to 5,241 participants. Among studies with available data, mean age ranged from 55.5 to 64.5 years, BMI from 25.2 to 26.9 kg/m^2^, and mean dietary energy intake from 1929.0 to 3247.4 kcal/day. UPF intake was mainly assessed using validated semi-quantitative food frequency questionnaires and defined according to the NOVA classification; exposure units included g/day, servings/day, UPF weight share, and study-defined intake categories. The included case–control studies commonly adjusted for age, sex, BMI, total energy intake, smoking, physical activity, education, alcohol consumption, family history of colorectal cancer where relevant, and other study-specific confounders.

Cancer outcomes were analysed separately by study design. Among cohort studies, five reported colorectal cancer; three reported colon, proximal colon, and distal colon cancers; four reported rectal cancer; two reported oesophageal adenocarcinoma, oesophageal squamous cell carcinoma, gastric cardia cancer, and non-cardia gastric cancer; three reported liver cancer; two reported hepatocellular carcinoma; and three reported pancreatic cancer. Among case–control studies, four reported colorectal cancer, two reported colon cancer, and two reported rectal cancer. Agreement between the two reviewers for data extraction was substantial (kappa = 0.774).

### Overall association between UPF intake and digestive system cancer risk

**Figure 2:**
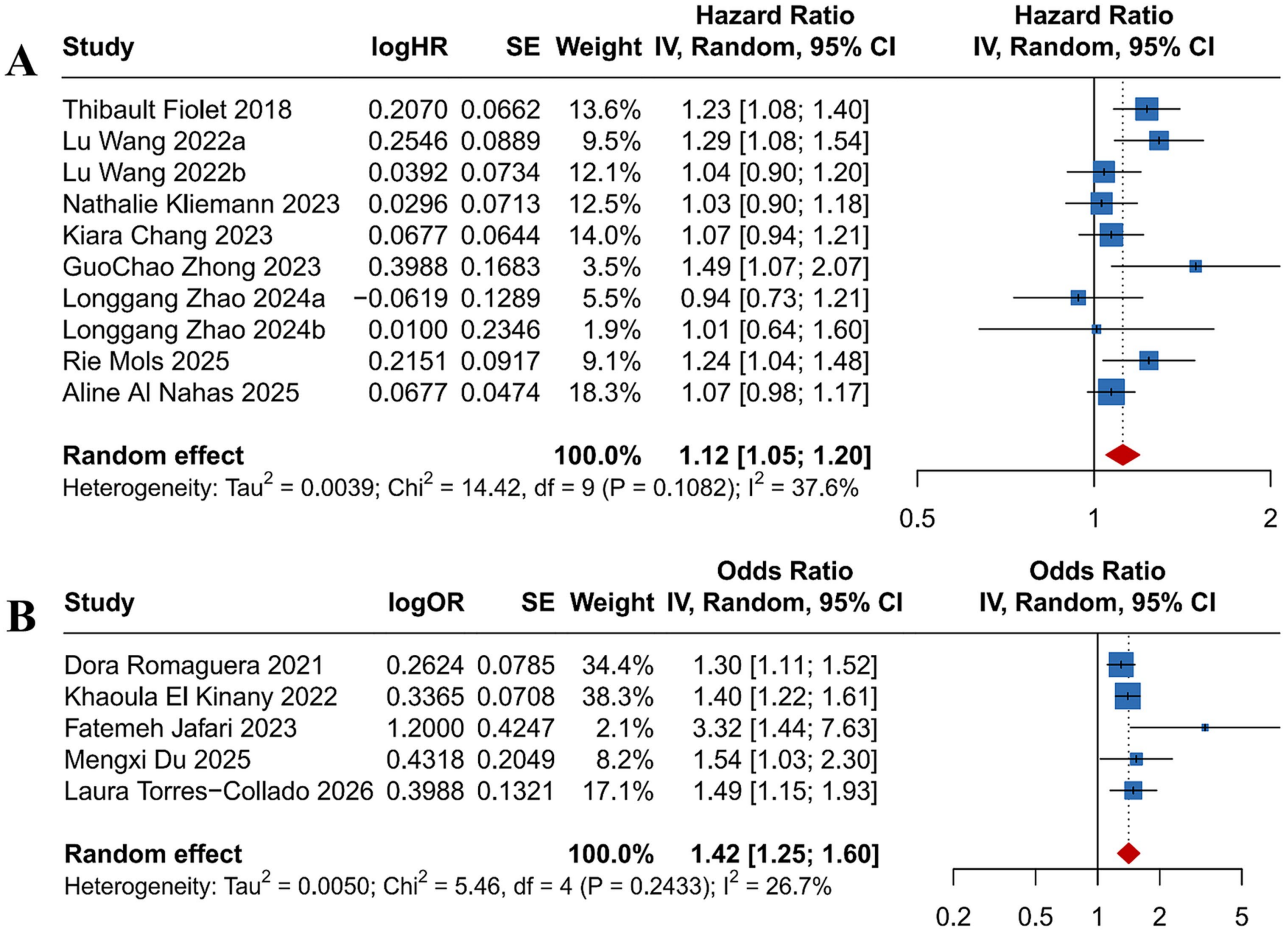
Overall association between ultra-processed food intake and digestive system cancer risk. Forest plots showing the association between the highest versus lowest ultra-processed food intake categories and digestive system cancer risk in **(A)** prospective cohort studies and **(B)** case–control studies.

Overall, when the highest UPF intake category was compared with the lowest category, cohort studies showed a 12% higher risk of digestive system cancers, whereas case–control studies showed 42% higher odds. In the cohort analysis, nine prospective cohort studies evaluated the association between UPF intake and digestive system cancer risk. The random-effects model produced a pooled HR of 1.12 (95% CI, 1.05–1.20), corresponding to a 12% higher risk of digestive system cancers (Figure 2A). Heterogeneity was low to moderate across cohort studies (*I*^2^ = 37.6%, *τ*^2^ = 0.0039, *p* = 0.1082).

In the case–control analysis, five studies evaluated this association. The pooled OR was 1.42 (95% CI: 1.25–1.60), corresponding to 42% higher odds of digestive system cancers (Figure 2B). Heterogeneity was mild across case–control studies (*I*^2^ = 26.7%, *τ*^2^ = 0.0050, *p* = 0.2433).

### Association between UPF intake and site-specific digestive system cancer risk

**Figure 3:**
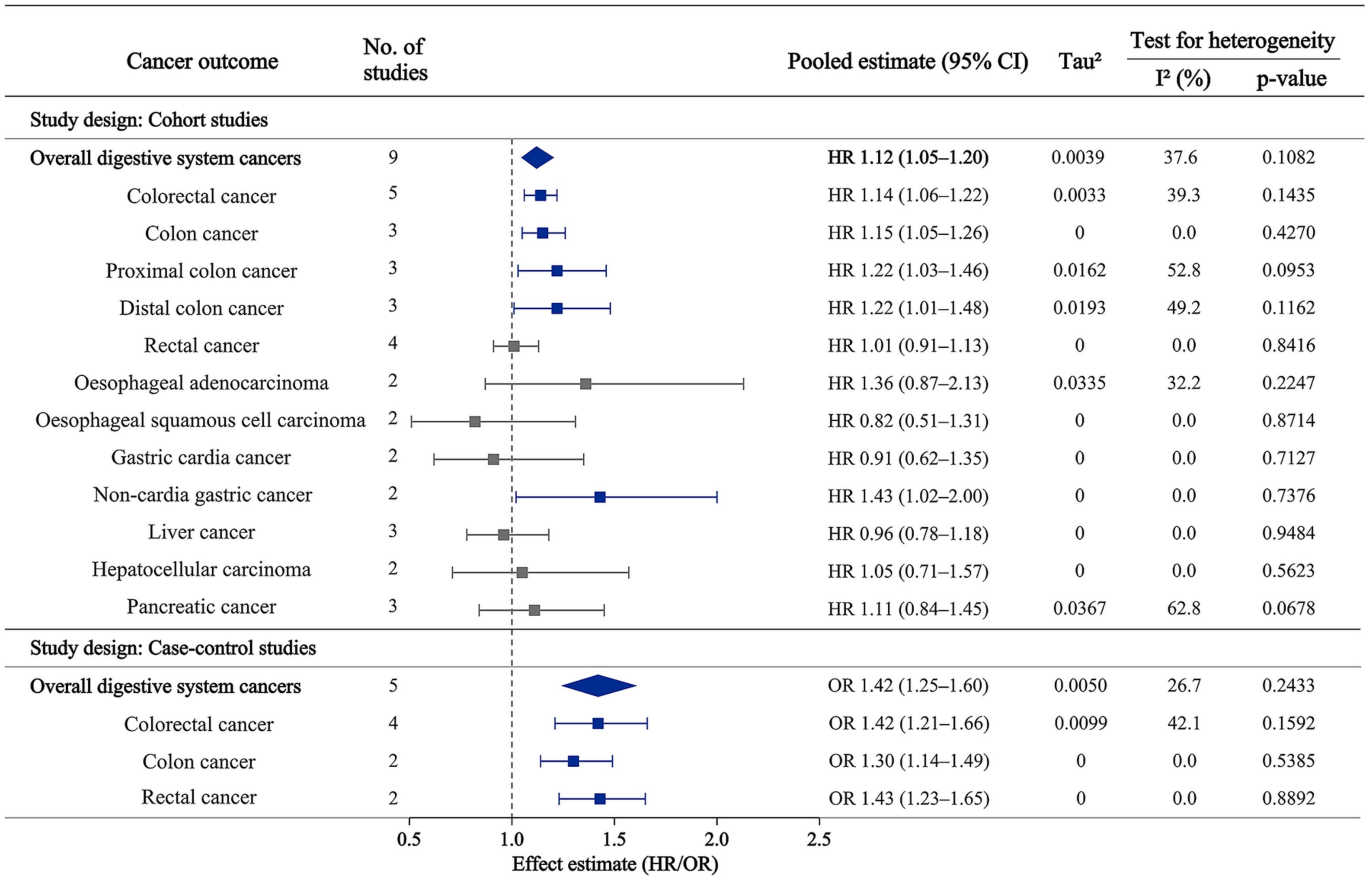
Site-specific associations between ultra-processed food intake and digestive system cancer risk. Pooled associations between the highest versus lowest ultra-processed food intake categories and site-specific digestive system cancer risk, stratified by study design.

We further analysed associations between UPF intake and site-specific digestive system cancer risk separately by study design (Figure 3; Supplementary Table S2; Supplementary Figures S1–S15). In cohort studies, higher UPF intake was associated with higher risks of colorectal cancer (HR = 1.14, 95% CI: 1.06–1.22), colon cancer (HR = 1.15, 95% CI: 1.05–1.26), proximal colon cancer (HR = 1.22, 95% CI: 1.03–1.46), distal colon cancer (HR = 1.22, 95% CI: 1.01–1.48), and non-cardia gastric cancer (HR = 1.43, 95% CI: 1.02–2.00). In contrast, pooled estimates for rectal cancer, oesophageal adenocarcinoma, oesophageal squamous cell carcinoma, gastric cardia cancer, liver cancer, hepatocellular carcinoma, and pancreatic cancer were not statistically significant. Between-study heterogeneity was generally low for most site-specific outcomes, except for proximal colon cancer, distal colon cancer, and pancreatic cancer, which showed moderate heterogeneity.

In case–control studies, higher UPF intake was associated with higher odds of colorectal cancer (OR = 1.42, 95% CI: 1.21–1.66), colon cancer (OR = 1.30, 95% CI: 1.14–1.49), and rectal cancer (OR = 1.43, 95% CI: 1.23–1.65), with low to moderate heterogeneity across site-specific outcomes. Overall, site-specific analyses suggested relatively consistent positive associations between UPF intake and colorectal and colon cancer outcomes, whereas evidence for other digestive system cancers, including oesophageal adenocarcinoma, oesophageal squamous cell carcinoma, gastric cardia cancer, liver cancer, hepatocellular carcinoma, and pancreatic cancer, remained limited.

### Association between UPF subcategories and digestive system cancer risk in cohort studies

In cohort studies, we further evaluated associations between UPF subcategories and digestive system cancer risk (Supplementary Table S4; Supplementary Figures S16–S20). Higher intake of ultra-processed meat/protein products was associated with a higher risk of digestive system cancers (HR = 1.33, 95% CI: 1.15–1.53), with moderate heterogeneity (*I*^2^ = 44.3%). In contrast, for UPF beverages (HR = 1.04, 95% CI: 0.90–1.19), ready-to-eat or ready-to-heat mixed dishes (HR = 1.15, 95% CI: 0.97–1.38), grain/bread products (HR = 1.13, 95% CI: 0.98–1.31), and sweets/desserts (HR = 1.04, 95% CI: 0.95–1.15), point estimates were above the null but were not statistically significant.

### Subgroup analyses and meta-regression analyses

**Figure 4:**
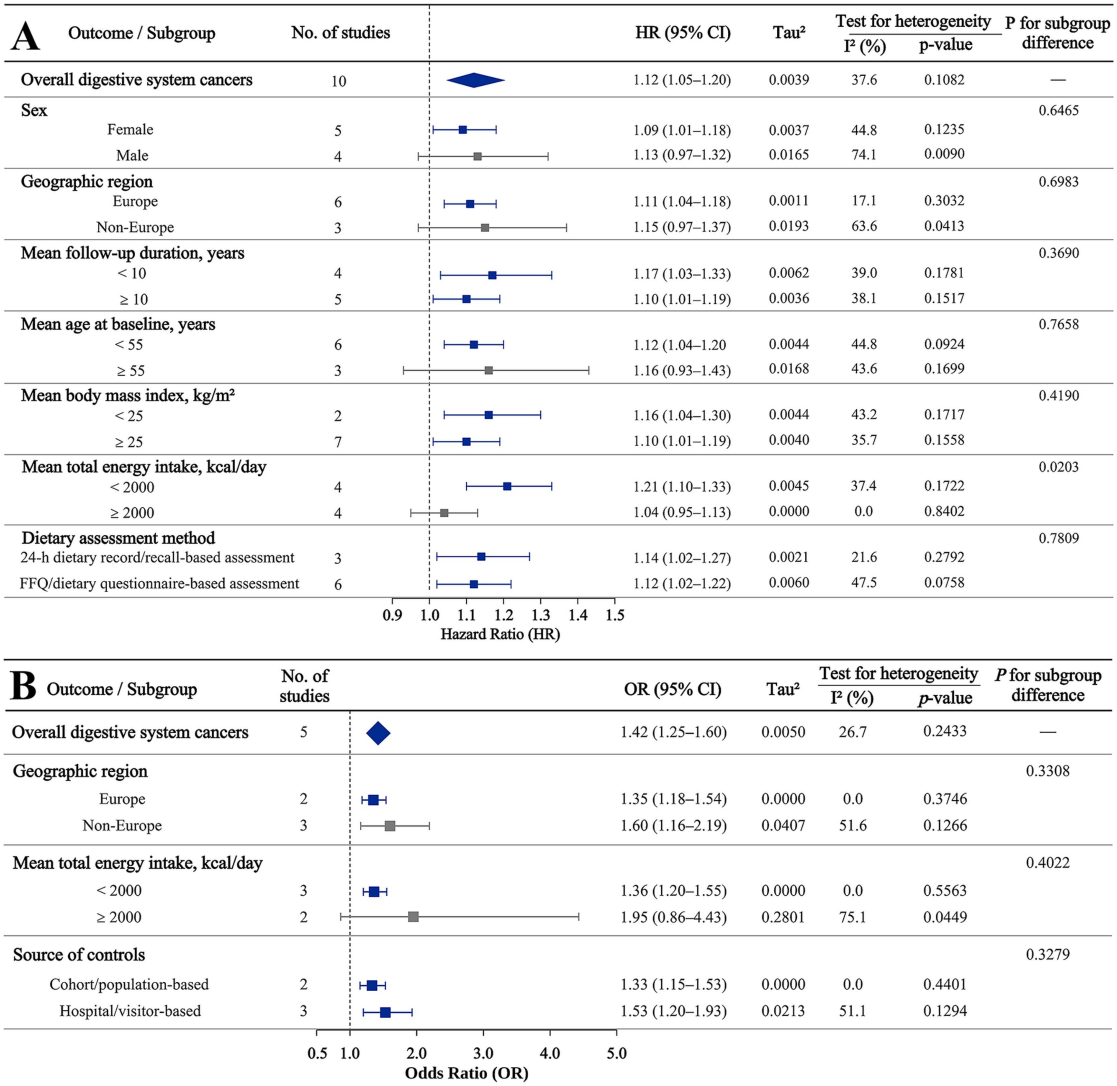
Subgroup analyses of the association between ultra-processed food intake and digestive system cancer risk. Subgroup analyses of the association between the highest versus lowest ultra-processed food intake categories and digestive system cancer risk in **(A)** prospective cohort studies and **(B)** case–control studies.

To evaluate the influence of study characteristics on pooled estimates, subgroup analyses were conducted separately for cohort and case–control studies (Figure 4; Supplementary Tables S5–S6; Supplementary Figures S21–S30). In the cohort studies, the association between higher UPF intake and digestive system cancer risk was directionally consistent across most subgroups. After stratification by sex, geographic region, follow-up duration, baseline age, BMI, mean total energy intake, and dietary assessment method, most subgroup estimates were above the null. Statistically significant associations were observed in female populations, European studies, both follow-up duration strata, studies with baseline age <55 years, both BMI strata, and studies using 24-h dietary records/recalls or FFQs/dietary questionnaires. After stratification by mean total energy intake, studies with <2000 kcal/day showed a significant association (HR = 1.21, 95% CI: 1.10–1.33), whereas those with ≥2000 kcal/day did not (HR = 1.04, 95% CI: 0.95–1.13), with a significant subgroup difference (*p* for subgroup difference = 0.0203).

In the case–control studies, subgroup analyses were generally consistent with the main analysis. After stratification by geographic region, mean total energy intake, and control source, most subgroup estimates suggested higher odds of digestive system cancers with higher UPF intake. The direction of association was consistent in European and non-European studies, and significant associations were observed in studies using cohort/population-based or hospital/visitor-based controls. When stratified by mean total energy intake, only the <2000 kcal/day subgroup was statistically significant (OR = 1.36, 95% CI: 1.20–1.55), whereas the ≥2000 kcal/day subgroup was not.

Because stratification by mean total energy intake showed a significant subgroup difference in cohort studies, exploratory meta-regression analyses were conducted. We examined factors that might influence pooled estimates, including subgroup characteristics, UPF exposure units, main-model covariate adjustments, and the number of adjusted covariates. None of these factors significantly explained between-study variation in effect estimates, indicating that the subgroup difference by mean total energy intake was not corroborated by meta-regression (Supplementary Table S7). Overall, the association between higher UPF intake and digestive system cancer risk remained broadly consistent across most study characteristics and model adjustment factors.

### Dose–response analysis

Because UPF intake was measured using different units across studies and dose–response data were limited, continuous exposure and dose–response analyses were conducted separately by exposure unit. UPF weight share (%g/day) was used as the primary analytical unit because it reflects the relative contribution of UPF to overall dietary intake. We first pooled cohort studies that directly reported continuous effect estimates. Each 10-percentage-point increase in UPF weight share was associated with a higher risk of digestive system cancers (HR = 1.07, 95% CI: 1.02–1.13), consistent with the linear trend observed in subsequent dose–response models (Supplementary Figure S31).

**Figure 5:**
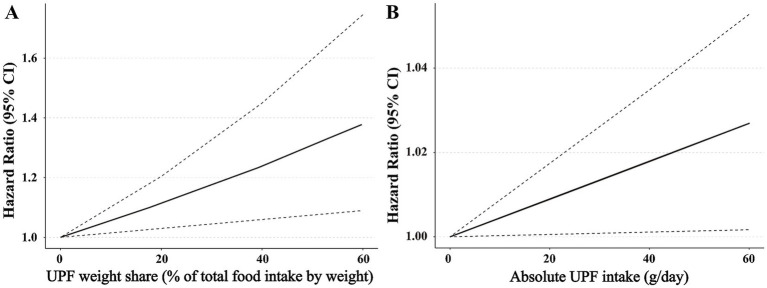
Linear dose–response associations between ultra-processed food intake and digestive system cancer risk. Linear dose–response associations of **(A)** ultra-processed food weight share and **(B)** absolute ultra-processed food intake with digestive system cancer risk. Solid lines represent pooled hazard ratios, and dashed lines indicate 95% confidence intervals.

In the primary dose–response analysis using UPF weight share (%g/day), higher UPF intake was associated with a higher risk of digestive system cancers (HR = 1.16, 95% CI: 1.04–1.31; Supplementary Figure S32). Linear dose–response analysis showed that each 10-percentage-point increase in UPF weight share was associated with an approximately 6% higher risk of digestive system cancers (HR = 1.06, 95% CI: 1.01–1.10; Figure 5A). The test for non-linearity was significant (*p* for non-linearity = 0.0046), suggesting that the association may not be strictly linear. The non-linear curve showed that risk generally increased with higher UPF weight share, with a higher point estimate around 40% intake. However, the confidence intervals were wide and partly crossed the null at both lower and higher exposure ranges. Therefore, this curve should be interpreted as suggesting a possible non-linear pattern rather than evidence of a reliable threshold. The current data do not allow identification of a clear risk threshold or a safe level of UPF intake, as detailed in the Supplementary Interpretation.

In the supplementary dose–response analysis using g/day, higher absolute UPF intake was also associated with a higher risk of digestive system cancers (HR = 1.21, 95% CI: 1.02–1.43; Supplementary Figure S33). The linear model showed that each 100 g/day increase in UPF intake was associated with an approximately 4.5% higher risk of digestive system cancers (HR = 1.045, 95% CI: 1.003–1.089; *p* = 0.036; Figure 5B). No significant non-linear association was observed (*p* for non-linearity = 0.1604), suggesting that, within the available data range, the association was compatible with an approximately linear trend.

### Publication bias and sensitivity analyses

Visual inspection of funnel plots did not suggest obvious asymmetry, and Egger’s test did not indicate significant publication bias (Supplementary Figures S34, S35). Leave-one-out sensitivity analyses showed that sequential exclusion of individual studies did not materially alter the pooled estimates for the association between UPF intake and digestive system cancer risk, suggesting that the overall findings were stable (Supplementary Figures S36, S37). Given the clearest positive association observed for ultra-processed meat/protein products, an additional exploratory leave-one-out sensitivity analysis confirmed the robustness of this subcategory finding, with the association remaining statistically significant after sequential exclusion of individual effect estimates (Supplementary Figure S38).

### Quality assessment and certainty of evidence

Methodological quality was assessed separately for the nine prospective cohort studies and five case–control studies using the NOS; all included studies were rated as high quality (Supplementary Tables S8, S9). In addition, the certainty of evidence for the main pooled and site-specific cancer outcomes was assessed using the GRADE framework. Because all included studies were observational, the initial certainty of evidence for each outcome was rated as low and then adjusted according to risk of bias, inconsistency, indirectness, imprecision, potential publication bias, and dose–response relationships. In cohort studies, the certainty of evidence for overall digestive system cancer risk was rated as moderate, whereas most site-specific outcomes were rated as low or very low; evidence from case–control studies was generally rated as low certainty (Supplementary Table S10). Agreement between the two reviewers for study quality assessment was almost perfect (kappa = 0.879).

## Discussion

This systematic review and meta-analysis synthesised data from 14 observational studies rated as high quality and evaluated associations between UPF intake and digestive system cancer risk, including dose–response relationships. Higher UPF intake was associated with a 12% higher risk in cohort studies and 42% higher odds in case–control studies. Site-specific analyses showed that higher UPF intake was associated with a higher risk of colorectal cancer, particularly colon cancer and its subsites, whereas pooled estimates for rectal cancer, oesophageal cancer subtypes, liver cancer, and pancreatic cancer were not statistically significant. Dose–response analyses were consistent with the main findings, showing that each 10-percentage-point increase in UPF weight share and each 100 g/day increase in UPF intake were associated with higher digestive system cancer risk, suggesting an exposure–response gradient.

Differences in pooled estimates between cohort and case–control studies may reflect differences in design and bias structure. Cohort studies assessed UPF intake before cancer occurrence, providing a clearer temporal sequence for evaluating associations between long-term dietary exposure and cancer incidence. In contrast, although case–control studies broadened the evidence base, they are more susceptible to recall and selection biases, particularly when dietary exposure is assessed retrospectively using FFQs after diagnosis, because cases may be more likely to recall or report unhealthy dietary behaviours. In addition, hospital- or visitor-based controls may not adequately represent UPF intake distributions in the source population. Therefore, the larger OR observed in case–control studies may partly reflect design-related biases and does not necessarily indicate a stronger underlying association than that observed in cohort studies. By pooling the two study designs separately, we avoided directly combining different effect measures and bias structures, thereby improving the interpretability of the findings.

Site-specific analyses suggested that the association between UPF intake and digestive system cancer risk may differ across cancer sites. In cohort studies, higher UPF intake was associated with a higher risk of colorectal cancer overall, and this association was more pronounced for colon cancer and its subsites, whereas the pooled estimate for rectal cancer did not reach statistical significance. Colorectal cancer shows substantial heterogeneity across anatomical sites, including differences in tumour-associated microbiota, bile acid-related metabolism, as well as immune and inflammatory pathways, risk-factor profiles, and molecular pathological features such as MSI, CIMP, and BRAF status (46–49). These differences may contribute to site-specific susceptibility to diet- and food processing-related exposures. In contrast, no statistically significant associations were observed for oesophageal cancer subtypes, gastric cardia cancer, liver cancer, hepatocellular carcinoma, or pancreatic cancer. This may reflect the small number of studies, differences in cancer aetiology, inconsistent exposure definitions, and limited statistical power. Therefore, these findings should not be interpreted as definitive evidence of no association, but rather as reflecting limitations in the current evidence base, requiring validation in large prospective studies.

Subgroup analyses showed that the association between higher UPF intake and digestive system cancer risk was generally directionally consistent across most study characteristics, although statistical significance varied across subgroups. Statistical significance within a subgroup may be influenced by the number of studies, sample size, and estimate precision, and does not necessarily indicate genuine effect modification. For example, in sex-stratified analyses, studies in female populations were statistically significant, whereas those in male populations were not; however, the point estimate in men (HR = 1.13) was not lower than that in women (HR = 1.09), suggesting that the difference was more likely due to wider confidence intervals or fewer studies, rather than indicating that UPF intake was associated with risk only in women (50, 51). Similarly, differences across geographic region, age, and mean total energy intake strata may have been jointly influenced by study source, dietary assessment method, UPF exposure unit, and cancer-site composition. Although stratification by mean total energy intake suggested a potential subgroup difference, exploratory meta-regression did not support it as a stable source of between-study heterogeneity. Moreover, mean total energy intake may partly proxy for unmeasured factors such as overall diet quality, socioeconomic conditions, health-care access, or cancer screening behaviour, rather than representing a true effect modifier. Therefore, the subgroup results mainly indicate broad consistency of the main findings across study characteristics, but are insufficient to identify definitive effect modifiers.

Among UPF subcategories, a statistically significant association with digestive system cancer risk was observed for ultra-processed meat/protein products, whereas other UPF subcategories showed point estimates above the null but did not reach statistical significance, suggesting that UPF is not a homogeneous dietary exposure and that different subcategories may have distinct risk profiles. Previous prospective studies have also suggested that associations between UPF subcategories and cancer risk may vary. In three US cohorts, Wang et al. (35) reported that sugar-sweetened beverages and ready-to-eat ultra-processed meat, poultry, and seafood products were associated with a higher risk of colorectal cancer in men. In the PLCO cohort, Zhong et al. (36) reported that intake of ultra-processed grains and meat was associated with a higher risk of pancreatic cancer. However, these findings were largely derived from analyses of specific cancer sites, selected populations, or sex-stratified subgroups, whereas our subcategory analysis pooled estimates from multiple cohort studies using overall digestive system cancer as the outcome, yielding a more conservative summary estimate. Mechanistically, ultra-processed meat/protein products may capture both “ultra-processed” and “processed meat” attributes. Processed meat has been classified by the IARC as carcinogenic to humans, with proposed mechanisms involving N-nitroso compounds, polycyclic aromatic hydrocarbons, heterocyclic amines, and haem iron-related oxidative damage (52). More broadly, UPF is a complex exposure, and its carcinogenic potential may involve multiple pathways, including poorer dietary quality, altered food matrices, additive exposure, increased energy intake, obesity-related metabolic abnormalities, and changes in the gut microenvironment. A randomised crossover feeding trial showed that an ultra-processed diet increased energy intake and short-term weight gain (13); the associated obesity, insulin resistance, and chronic low-grade inflammation may contribute to colorectal carcinogenesis through the FABP5/PPARD/CPT1A axis, inflammatory mediators, and oxidative stress (53–55). In addition, additives and processing-related components in industrial UPF formulations may contribute to chronic gastrointestinal inflammation through gut microbiota- and mucosal barrier-related pathways (56). Experimental studies have also suggested that certain dietary emulsifiers and artificial sweeteners may be involved in metabolic dysregulation and intestinal tumourigenesis through effects on the gut microbiota, mucosal barrier, and inflammatory responses (57–59). These mechanisms may partly explain why associations were more consistently observed for colorectal and colon cancer outcomes and for ultra-processed meat/protein products. However, further prospective studies incorporating gut microbiota profiling, inflammatory markers, metabolomics, and molecular tumour subtyping are needed for validation.

The dose–response results provided additional support for the main findings. Compared with highest-versus-lowest comparisons, dose–response analyses provide more detailed information on exposure gradients. In this study, each 10-percentage-point increase in UPF weight share was associated with a modestly higher risk of digestive system cancers; the supplementary analysis using g/day showed a similar linear trend. These findings are consistent with a graded association across the available exposure range, although the observational nature of the evidence precludes causal interpretation. However, because confidence intervals were wide in the higher exposure range of the non-linear analysis, the current evidence is insufficient to define a specific safe intake level or clear risk threshold.

### Comparison with previous studies

Previous systematic reviews and meta-analyses of UPF intake and cancer risk have mainly focused on selected cancer types. A pan-cancer meta-analysis showed that higher UPF intake was associated with higher risks of several site-specific cancers, particularly colorectal, colon, and breast cancers, but did not specifically focus on the digestive system cancer spectrum (60). An umbrella review also suggested that UPF intake was associated with multiple adverse health outcomes, with relatively consistent evidence for colorectal cancer (61). A previous meta-analysis of prospective cohort studies evaluated UPF intake and gastrointestinal cancer risk; however, it primarily pooled estimates by cancer type or anatomical subsite and did not provide an overall estimate for the full digestive system cancer spectrum (9). In addition, meta-analyses focusing on colorectal cancer have shown that higher UPF intake is associated with higher colorectal cancer risk, but have provided limited exploration of dose–response relationships and differences across digestive system cancer types (62).

Compared with previous studies, the present study differs in several respects. First, this study did not focus solely on overall cancer or colorectal cancer, but systematically evaluated the full spectrum of digestive system cancers. Second, this study included both cohort and case–control studies and pooled HRs and ORs separately by study design, thereby avoiding interpretive problems arising from directly combining different study designs, effect measures, and bias structures. Third, this study conducted site-specific, subgroup, UPF subcategory, continuous exposure, and dose–response analyses, and incorporated NOS and GRADE assessments to evaluate methodological quality and certainty of evidence, thereby improving the transparency of evidence interpretation across outcomes. Overall, consistent with previous evidence linking UPF intake to colorectal and colon cancer risk, this study further suggests that associations may vary across digestive system cancer sites and that ultra-processed meat/protein products may represent a relevant contributing subcategory.

### Strengths and limitations

This study has several strengths. First, this study systematically evaluated UPF intake across the full spectrum of digestive system cancers, rather than focusing only on overall cancer, colorectal cancer, or selected gastrointestinal outcomes. Second, cohort and case–control studies were analysed separately, with HRs and ORs pooled according to study design, thereby avoiding interpretive issues from combining different designs, effect measures, and bias structures. Third, digestive system cancers were stratified by anatomical site and subtype, and continuous exposure, dose–response, and UPF subcategory analyses were performed, allowing assessment of UPF-related associations across cancer sites, exposure gradients, and food categories. Finally, methodological quality and certainty of evidence were assessed using the NOS and the GRADE framework, respectively; sensitivity analyses, publication bias assessment, subgroup analyses, and meta-regression were conducted to evaluate result stability and potential sources of heterogeneity.

This study also has several limitations. First, all included studies were observational, and residual or unmeasured confounding could not be excluded, including confounding by overall diet quality, socioeconomic status, cancer screening behaviour, *Helicobacter pylori* infection, antibiotic use, and other lifestyle factors. Second, UPF classification depended on dietary assessment tools, food databases, and NOVA-based classification algorithms used in the original studies, and exposure misclassification may have occurred across countries and cohorts. In addition, inconsistent UPF intake units limited the number of studies available for continuous exposure and dose–response analyses. Third, some site-specific cancer outcomes and UPF subcategory analyses were based on few studies and had wide confidence intervals and low GRADE certainty, and should therefore be interpreted cautiously. Fourth, case–control studies may have been affected by recall and selection biases, particularly those using hospital- or visitor-based controls. Finally, the available evidence was mainly derived from Europe, North America, and other high-income settings, where UPF composition, food industrialisation, dietary patterns, and cancer screening practices may differ from those in other regions; therefore, generalisability to low- and middle-income countries may be limited. Accordingly, future prospective studies should prioritise repeated dietary assessment using validated NOVA-based algorithms, integration of gut microbiota, inflammatory, and metabolic biomarkers, refined cancer subtyping, and inclusion of populations from low- and middle-income countries where UPF consumption is increasing.

## Conclusion

This systematic review and meta-analysis showed that higher UPF intake was associated with a higher risk of digestive system cancers, with relatively consistent evidence for colorectal and colon cancers. Ultra-processed meat/protein products may represent a relevant contributing subcategory, and continuous exposure and dose–response analyses supported an exposure–response gradient. However, the current dose–response evidence does not allow identification of a reliable risk threshold. Because the available evidence was mainly derived from observational studies and remains limited for some site-specific cancers and UPF subcategories, these findings should be interpreted as supportive rather than causal. Further high-quality prospective studies incorporating standardised UPF classification, repeated dietary assessment, mechanistic biomarkers, and refined cancer subtyping are needed to validate these associations.

## Data Availability

The original contributions presented in the study are included in the article/Supplementary material, further inquiries can be directed to the corresponding authors.
