# Supplementary material for: Ultra-processed food intake and risk of digestive system cancers: a systematic review and dose–response meta-analysis
Source: Front Nutr. 2026 Jul 15;13:1901660. doi: 10.3389/fnut.2026.1901660 (PMC13414877; doi:10.3389/fnut.2026.1901660)
Supplement: Supplementary file 1 [file Table_1.docx]

**Supplementary catalogue**

[Search strategies 1](#_Toc25006)

[Supplementary Interpretation: Supplementary Interpretation: Explanation of the Non-linear Dose–Response Association Between UPF Intake and Digestive System Cancer Risk 4](#_Toc774)

[Supplementary Figure S1. Forest plot of the association between UPF intake and colorectal cancer risk in cohort studies. 6](#_Toc3174)

[Supplementary Figure S2. Forest plot of the association between UPF intake and colon cancer risk in cohort studies. 7](#_Toc22461)

[Supplementary Figure S3. Forest plot of the association between UPF intake and proximal colon cancer risk in cohort studies. 8](#_Toc28737)

[Supplementary Figure S4. Forest plot of the association between UPF intake and distal colon cancer risk in cohort studies. 9](#_Toc25672)

[Supplementary Figure S5. Forest plot of the association between UPF intake and rectal cancer risk in cohort studies. 10](#_Toc2026)

[Supplementary Figure S6. Forest plot of the association between UPF intake and oesophageal adenocarcinoma risk in cohort studies. 11](#_Toc13459)

[Supplementary Figure S7. Forest plot of the association between UPF intake and oesophageal squamous cell carcinoma risk in cohort studies. 12](#_Toc25565)

[Supplementary Figure S8. Forest plot of the association between UPF intake and gastric cardia cancer risk in cohort studies. 13](#_Toc607)

[Supplementary Figure S9. Forest plot of the association between UPF intake and non-cardia gastric cancer risk in cohort studies. 14](#_Toc32040)

[Supplementary Figure S10. Forest plot of the association between UPF intake and liver cancer risk in cohort studies. 15](#_Toc14221)

[Supplementary Figure S11. Forest plot of the association between UPF intake and hepatocellular carcinoma risk in cohort studies. 16](#_Toc16140)

[Supplementary Figure S12. Forest plot of the association between UPF intake and pancreatic cancer risk in cohort studies. 17](#_Toc8121)

[Supplementary Figure S13. Forest plot of the association between UPF intake and colorectal cancer risk in case-control studies. 18](#_Toc1703)

[Supplementary Figure S14. Forest plot of the association between UPF intake and colon cancer risk in case-control studies. 19](#_Toc11180)

[Supplementary Figure S15. Forest plot of the association between UPF intake and rectal cancer risk in case-control studies. 20](#_Toc4383)

[Supplementary Figure S16. Forest plot of the association between UPF meat/protein product intake and digestive system cancer risk in cohort studies. 21](#_Toc32116)

[Supplementary Figure S17. Forest plot of the association between UPF beverage intake and digestive system cancer risk in cohort studies. 22](#_Toc21467)

[Supplementary Figure S18. Forest plot of the association between UPF ready meal intake and digestive system cancer risk in cohort studies. 23](#_Toc27169)

[Supplementary Figure S19. Forest plot of the association between UPF grain/bread product intake and digestive system cancer risk in cohort studies. 24](#_Toc22479)

[Supplementary Figure S20. Forest plot of the association between UPF sweet snack/dessert intake and digestive system cancer risk in cohort studies. 25](#_Toc18035)

[Supplementary Figure S21. Meta-analysis of the association between UPF intake and digestive system cancer risk in cohort studies stratified by sex. 26](#_Toc14315)

[Supplementary Figure S22. Meta-analysis of the association between UPF intake and digestive system cancer risk in cohort studies stratified by geographic region. 27](#_Toc15877)

[Supplementary Figure S23. Meta-analysis of the association between UPF intake and digestive system cancer risk in cohort studies stratified by mean follow-up duration. 28](#_Toc9252)

[Supplementary Figure S24. Meta-analysis of the association between UPF intake and digestive system cancer risk in cohort studies stratified by mean age at baseline. 29](#_Toc12466)

[Supplementary Figure S25. Meta-analysis of the association between UPF intake and digestive system cancer risk in cohort studies stratified by mean body mass index. 30](#_Toc5560)

[Supplementary Figure S26. Meta-analysis of the association between UPF intake and digestive system cancer risk in cohort studies stratified by mean total energy intake. 31](#_Toc12362)

[Supplementary Figure S27. Meta-analysis of the association between UPF intake and digestive system cancer risk in cohort studies stratified by dietary assessment method. 32](#_Toc1686)

[Supplementary Figure S28. Meta-analysis of the association between UPF intake and digestive system cancer risk in case-control studies stratified by geographic region. 33](#_Toc18646)

[Supplementary Figure S29. Meta-analysis of the association between UPF intake and digestive system cancer risk in case-control studies stratified by mean total energy intake. 34](#_Toc19738)

[Supplementary Figure S30. Meta-analysis of the association between UPF intake and digestive system cancer risk in case-control studies stratified by source of controls. 35](#_Toc20466)

[Supplementary Figure S31. Forest plot of the association between each 10-percentage-point increase in UPF weight share and digestive system cancer risk in cohort studies. 36](#_Toc1651)

[Supplementary Figure S32. Forest plot of study-specific summary estimates for the dose–response association between UPF weight share (%g/day) and digestive system cancer risk in cohort studies. 37](#_Toc7177)

[Supplementary Figure S33. Forest plot of study-specific summary estimates for the dose–response association between absolute UPF intake (g/day) and digestive system cancer risk in cohort studies. 38](#_Toc3436)

[Supplementary Figure S34. Funnel plot and Egger’s test for publication bias in the association between UPF intake and digestive system cancer risk in cohort studies. 39](#_Toc19070)

[Supplementary Figure S35. Funnel plot and Egger’s test for publication bias in the association between UPF intake and digestive system cancer risk in case-control studies. 40](#_Toc10368)

[Supplementary Figure S36. Leave-one-out sensitivity analysis of the association between UPF intake and digestive system cancer risk in cohort studies. 41](#_Toc13316)

[Supplementary Figure S37. Leave-one-out sensitivity analysis of the association between UPF intake and digestive system cancer risk in case-control studies. 42](#_Toc12620)

[Supplementary Figure S38. Leave-one-out sensitivity analysis of the association between UPF meat/protein product intake and digestive system cancer risk in cohort studies. 43](#_Toc26794)

[Supplementary Table S1. The treatment of duplicate data. 44](#_Toc18659)

[Supplementary Table S2. Summary of meta-analysis results for the association between UPF intake and digestive system cancer risk. 47](#_Toc6509)

[Supplementary Table S3. Literature was excluded after full-text review for failing to meet the inclusion criteria. 48](#_Toc1948)

[Supplementary Table S4. Meta-analysis results for the association between UPF subcategory intake and digestive system cancer risk in cohort studies. 52](#_Toc17239)

[Supplementary Table S5. Subgroup analyses of the association between UPF intake and digestive system cancer risk in cohort studies. 53](#_Toc19520)

[Supplementary Table S6. Subgroup analyses of the association between UPF intake and digestive system cancer risk in case-control studies. 54](#_Toc20580)

[Supplementary Table S7. Meta-regression analyses of study-level modifiers for the association between UPF intake and digestive system cancer risk. 55](#_Toc11724)

[Supplementary Table S8. Newcastle–Ottawa Scale quality assessment of cohort studies. 56](#_Toc17609)

[Supplementary Table S9. Newcastle–Ottawa Scale quality assessment of case-control studies. 57](#_Toc27683)

[Supplementary Table S10. GRADE certainty-of-evidence assessment for the association between UPF intake and digestive system cancer risk. 58](#_Toc857)

# Search strategies

Medical subject headings (MeSH) and non-MeSH terms used to search relevant publications on the association between UPF and risk of Digestive System Cancers ^1^.

| **Database** | **Step** | **Terms** (Search strategy for the Meta-Analysis, up to May 1, 2026) | **Results** |
| --- | --- | --- | --- |
| **PubMed** | 1 | “Food, Processed” [Mesh] OR “Food, Processed” [Title/Abstract] OR “ultra-processed foods” [Title/Abstract] OR “ultra-processed food” [Title/Abstract] OR “ultraprocessed foods” [Title/Abstract] OR “ultraprocessed food” [Title/Abstract] OR “ultra processed foods” [Title/Abstract] OR “ultra processed food” [Title/Abstract] OR “ultraprocessed” [Title/Abstract] OR “ultra-processed” [Title/Abstract] OR “ultra processed” [Title/Abstract] OR “UPF” [Title/Abstract] OR “UPFs” [Title/Abstract] OR “NOVA classification” [Title/Abstract] OR “NOVA food classification” [Title/Abstract] OR “NOVA 4” [Title/Abstract] OR “NOVA group 4” [Title/Abstract] OR “food processing” [Title/Abstract] OR “processed foods” [Title/Abstract] OR “processed food” [Title/Abstract] |  |
|  | 2 | “Neoplasms” [Mesh] OR “Digestive System Neoplasms” [Mesh] OR “Gastrointestinal Neoplasms” [Mesh] OR “Neoplasms” [Title/Abstract] OR “Digestive System Neoplasms” [Title/Abstract] OR “Gastrointestinal Neoplasms” [Title/Abstract] OR “neoplasms” [Title/Abstract] OR “neoplasm” [Title/Abstract] OR “neoplasias” [Title/Abstract] OR “neoplasia” [Title/Abstract] OR “cancers” [Title/Abstract] OR “cancer” [Title/Abstract] OR “tumors” [Title/Abstract] OR “tumor” [Title/Abstract] OR “tumours” [Title/Abstract] OR “tumour” [Title/Abstract] OR “malignant” [Title/Abstract] OR “malignancy” [Title/Abstract] OR “malignancies” [Title/Abstract] OR “malignant neoplasms” [Title/Abstract] OR “malignant neoplasm” [Title/Abstract] OR “neoplasms, malignant” [Title/Abstract] OR “neoplasm, malignant” [Title/Abstract] OR “carcinomas” [Title/Abstract] OR “carcinoma” [Title/Abstract] OR “adenocarcinomas” [Title/Abstract] OR “adenocarcinoma” [Title/Abstract] |  |
|  | 3 | “Digestive System” [Mesh] OR “digestive system” [Title/Abstract] OR “digestive” [Title/Abstract] OR “digestive tract” [Title/Abstract] OR “gastrointestinal” [Title/Abstract] OR “gastro intestinal” [Title/Abstract] OR “GI tract” [Title/Abstract] OR “esophagus” [Title/Abstract] OR “oesophagus” [Title/Abstract] OR “esophageal” [Title/Abstract] OR “oesophageal” [Title/Abstract] OR “gastroesophageal” [Title/Abstract] OR “gastro-oesophageal” [Title/Abstract] OR “gastroesophageal junction” [Title/Abstract] OR “gastro-oesophageal junction” [Title/Abstract] OR “esophagogastric” [Title/Abstract] OR “oesophagogastric” [Title/Abstract] OR “GEJ” [Title/Abstract] OR “GOJ” [Title/Abstract] OR “stomach” [Title/Abstract] OR “gastric” [Title/Abstract] OR “intestinal” [Title/Abstract] OR “intestine” [Title/Abstract] OR “bowel” [Title/Abstract] OR “duodenal” [Title/Abstract] OR “jejunal” [Title/Abstract] OR “ileal” [Title/Abstract] OR “appendiceal” [Title/Abstract] OR “colon” [Title/Abstract] OR “colonic” [Title/Abstract] OR “colorectal” [Title/Abstract] OR “rectal” [Title/Abstract] OR “rectum” [Title/Abstract] OR “rectosigmoid” [Title/Abstract] OR “cecum” [Title/Abstract] OR “caecum” [Title/Abstract] OR “anal” [Title/Abstract] OR “anus” [Title/Abstract] OR “liver” [Title/Abstract] OR “hepatic” [Title/Abstract] OR “hepatocellular” [Title/Abstract] OR “hepatobiliary” [Title/Abstract] OR “hepato-biliary” [Title/Abstract] OR “hepatopancreatobiliary” [Title/Abstract] OR “hepato-pancreato-biliary” [Title/Abstract] OR “biliary” [Title/Abstract] OR “biliary tract” [Title/Abstract] OR “bile duct” [Title/Abstract] OR “bile ducts” [Title/Abstract] OR “cholangiocarcinoma” [Title/Abstract] OR “gallbladder” [Title/Abstract] OR “gall bladder” [Title/Abstract] OR “pancreas” [Title/Abstract] OR “pancreatic” [Title/Abstract] OR “ampullary” [Title/Abstract] OR “periampullary” [Title/Abstract] OR “ampulla of Vater” [Title/Abstract] |  |
|  | 4 | #1 AND #2 AND #3 | **384** |
| **Embase** | 1 | (‘Food**,** Processed’/exp OR ‘Food**,** Processed’:ab,ti,kw OR ‘ultra-processed foods’:ab,ti,kw OR ‘ultra-processed food’:ab,ti,kw OR ‘ultraprocessed foods’:ab,ti,kw OR ‘ultraprocessed food’:ab,ti,kw OR ‘ultra processed foods’:ab,ti,kw OR ‘ultra processed food’:ab,ti,kw OR ‘ultraprocessed’:ab,ti,kw OR ‘ultra-processed’:ab,ti,kw OR ‘ultra processed’:ab,ti,kw OR ‘UPF’:ab,ti,kw OR ‘UPFs’:ab,ti,kw OR ‘NOVA classification’:ab,ti,kw OR ‘NOVA food classification’:ab,ti,kw OR ‘NOVA 4’:ab,ti,kw OR ‘NOVA group 4’:ab,ti,kw OR ‘food processing’:ab,ti,kw OR ‘processed foods’:ab,ti,kw OR ‘processed food’:ab,ti,kw) |  |
|  | 2 | (‘Neoplasms’/exp OR ‘Digestive System Neoplasms’/exp OR ‘Gastrointestinal Neoplasms’/exp OR ‘Neoplasms’:ab,ti,kw OR ‘Digestive System Neoplasms’:ab,ti,kw OR ‘Gastrointestinal Neoplasms’:ab,ti,kw OR ‘neoplasms’:ab,ti,kw OR ‘neoplasm’:ab,ti,kw OR ‘neoplasias’:ab,ti,kw OR ‘neoplasia’:ab,ti,kw OR ‘cancers’:ab,ti,kw OR ‘cancer’:ab,ti,kw OR ‘tumors’:ab,ti,kw OR ‘tumor’:ab,ti,kw OR ‘tumours’:ab,ti,kw OR ‘tumour’:ab,ti,kw OR ‘malignant’:ab,ti,kw OR ‘malignancy’:ab,ti,kw OR ‘malignancies’:ab,ti,kw OR ‘malignant neoplasms’:ab,ti,kw OR ‘malignant neoplasm’:ab,ti,kw OR ‘neoplasms, malignant’:ab,ti,kw OR ‘neoplasm, malignant’:ab,ti,kw OR ‘carcinomas’:ab,ti,kw OR ‘carcinoma’:ab,ti,kw OR ‘adenocarcinomas’:ab,ti,kw OR ‘adenocarcinoma’:ab,ti,kw) |  |
|  | 3 | (‘Digestive System’/exp OR ‘digestive system’:ab,ti,kw OR ‘digestive’:ab,ti,kw OR ‘digestive tract’:ab,ti,kw OR ‘gastrointestinal’:ab,ti,kw OR ‘gastro intestinal’:ab,ti,kw OR ‘GI tract’:ab,ti,kw OR ‘esophagus’:ab,ti,kw OR ‘oesophagus’:ab,ti,kw OR ‘esophageal’:ab,ti,kw OR ‘oesophageal’:ab,ti,kw OR ‘gastroesophageal’:ab,ti,kw OR ‘gastro-oesophageal’:ab,ti,kw OR ‘gastroesophageal junction’:ab,ti,kw OR ‘gastro-oesophageal junction’:ab,ti,kw OR ‘esophagogastric’:ab,ti,kw OR ‘oesophagogastric’:ab,ti,kw OR ‘GEJ’:ab,ti,kw OR ‘GOJ’:ab,ti,kw OR ‘stomach’:ab,ti,kw OR ‘gastric’:ab,ti,kw OR ‘intestinal’:ab,ti,kw OR ‘intestine’:ab,ti,kw OR ‘bowel’:ab,ti,kw OR ‘duodenal’:ab,ti,kw OR ‘jejunal’:ab,ti,kw OR ‘ileal’:ab,ti,kw OR ‘appendiceal’:ab,ti,kw OR ‘colon’:ab,ti,kw OR ‘colonic’:ab,ti,kw OR ‘colorectal’:ab,ti,kw OR ‘rectal’:ab,ti,kw OR ‘rectum’:ab,ti,kw OR ‘rectosigmoid’:ab,ti,kw OR ‘cecum’:ab,ti,kw OR ‘caecum’:ab,ti,kw OR ‘anal’:ab,ti,kw OR ‘anus’:ab,ti,kw OR ‘liver’:ab,ti,kw OR ‘hepatic’:ab,ti,kw OR ‘hepatocellular’:ab,ti,kw OR ‘hepatobiliary’:ab,ti,kw OR ‘hepato-biliary’:ab,ti,kw OR ‘hepatopancreatobiliary’:ab,ti,kw OR ‘hepato-pancreato-biliary’:ab,ti,kw OR ‘biliary’:ab,ti,kw OR ‘biliary tract’:ab,ti,kw OR ‘bile duct’:ab,ti,kw OR ‘bile ducts’:ab,ti,kw OR ‘cholangiocarcinoma’:ab,ti,kw OR ‘gallbladder’:ab,ti,kw OR ‘gall bladder’:ab,ti,kw OR ‘pancreas’:ab,ti,kw OR ‘pancreatic’:ab,ti,kw OR ‘ampullary’:ab,ti,kw OR ‘periampullary’:ab,ti,kw OR ‘ampulla of Vater’:ab,ti,kw) |  |
|  | 4 | #1 AND #2 AND #3 | **1,436** |
| **Web of Science** | 1 | TS=(“Food, Processed” OR “ultra-processed foods” OR “ultra-processed food” OR “ultraprocessed foods” OR “ultraprocessed food” OR “ultra processed foods” OR “ultra processed food” OR “ultraprocessed” OR “ultra-processed” OR “ultra processed” OR “UPF” OR “UPFs” OR “NOVA classification” OR “NOVA food classification” OR “NOVA 4” OR “NOVA group 4” OR “food processing” OR “processed foods” OR “processed food”) |  |
|  | 2 | TS=(“Neoplasms” OR “Digestive System Neoplasms” OR “Gastrointestinal Neoplasms” OR “neoplasms” OR “neoplasm” OR “neoplasias” OR “neoplasia” OR “cancers” OR “cancer” OR “tumors” OR “tumor” OR “tumours” OR “tumour” OR “malignant” OR “malignancy” OR “malignancies” OR “malignant neoplasms” OR “malignant neoplasm” OR “neoplasms, malignant” OR “neoplasm, malignant” OR “carcinomas” OR “carcinoma” OR “adenocarcinomas” OR “adenocarcinoma”) |  |
|  | 3 | TS=(“digestive system” OR “digestive” OR “digestive tract” OR “gastrointestinal” OR “gastro intestinal” OR “GI tract” OR “esophagus” OR “oesophagus” OR “esophageal” OR “oesophageal” OR “gastroesophageal” OR “gastro-oesophageal” OR “gastroesophageal junction” OR “gastro-oesophageal junction” OR “esophagogastric” OR “oesophagogastric” OR “GEJ” OR “GOJ” OR “stomach” OR “gastric” OR “intestinal” OR “intestine” OR “bowel” OR “duodenal” OR “jejunal” OR “ileal” OR “appendiceal” OR “colon” OR “colonic” OR “colorectal” OR “rectal” OR “rectum” OR “rectosigmoid” OR “cecum” OR “caecum” OR “anal” OR “anus” OR “liver” OR “hepatic” OR “hepatocellular” OR “hepatobiliary” OR “hepato-biliary” OR “hepatopancreatobiliary” OR “hepato-pancreato-biliary” OR “biliary” OR “biliary tract” OR “bile duct” OR “bile ducts” OR “cholangiocarcinoma” OR “gallbladder” OR “gall bladder” OR “pancreas” OR “pancreatic” OR “ampullary” OR “periampullary” OR “ampulla of Vater”) |  |
|  | 4 | #1 AND #2 AND #3 | **500** |
| **Cochrane Library** | 1 | MeSH descriptor: [Food, Processed] explode all trees OR (“ultra-processed foods” OR “ultra-processed food” OR “ultraprocessed foods” OR “ultraprocessed food” OR “ultra processed foods” OR “ultra processed food” OR “ultraprocessed” OR “ultra-processed” OR “ultra processed” OR “UPF” OR “UPFs” OR “NOVA classification” OR “NOVA food classification” OR “NOVA 4” OR “NOVA group 4” OR “food processing” OR “processed foods” OR “processed food”):ti,ab,kw |  |
|  | 2 | MeSH descriptor: [Neoplasms] explode all trees OR MeSH descriptor: [Digestive System Neoplasms] explode all trees OR MeSH descriptor: [Gastrointestinal Neoplasms] explode all trees OR (“Neoplasms” OR “Digestive System Neoplasms” OR “Gastrointestinal Neoplasms” OR “neoplasms” OR “neoplasm” OR “neoplasias” OR “neoplasia” OR “cancers” OR “cancer” OR “tumors” OR “tumor” OR “tumours” OR “tumour” OR “malignant” OR “malignancy” OR “malignancies” OR “malignant neoplasms” OR “malignant neoplasm” OR “neoplasms, malignant” OR “neoplasm, malignant” OR “carcinomas” OR “carcinoma” OR “adenocarcinomas” OR “adenocarcinoma”):ti,ab,kw |  |
|  | 3 | MeSH descriptor: [Digestive System] explode all trees OR (“digestive system” OR “digestive” OR “digestive tract” OR “gastrointestinal” OR “gastro intestinal” OR “GI tract” OR “esophagus” OR “oesophagus” OR “esophageal” OR “oesophageal” OR “gastroesophageal” OR “gastro-oesophageal” OR “gastroesophageal junction” OR “gastro-oesophageal junction” OR “esophagogastric” OR “oesophagogastric” OR “GEJ” OR “GOJ” OR “stomach” OR “gastric” OR “intestinal” OR “intestine” OR “bowel” OR “duodenal” OR “jejunal” OR “ileal” OR “appendiceal” OR “colon” OR “colonic” OR “colorectal” OR “rectal” OR “rectum” OR “rectosigmoid” OR “cecum” OR “caecum” OR “anal” OR “anus” OR “liver” OR “hepatic” OR “hepatocellular” OR “hepatobiliary” OR “hepato-biliary” OR “hepatopancreatobiliary” OR “hepato-pancreato-biliary” OR “biliary” OR “biliary tract” OR “bile duct” OR “bile ducts” OR “cholangiocarcinoma” OR “gallbladder” OR “gall bladder” OR “pancreas” OR “pancreatic” OR “ampullary” OR “periampullary” OR “ampulla of Vater”):ti,ab,kw |  |
|  | 4 | #1 AND #2 AND #3 | **30** |
| **Total** |  |  | **2,350** |

^1^Two investigators (JYJ & ZSP) searched the online databases independently.

# Supplementary Interpretation: Supplementary Interpretation: Explanation of the Non-linear Dose–Response Association Between UPF Intake and Digestive System Cancer Risk


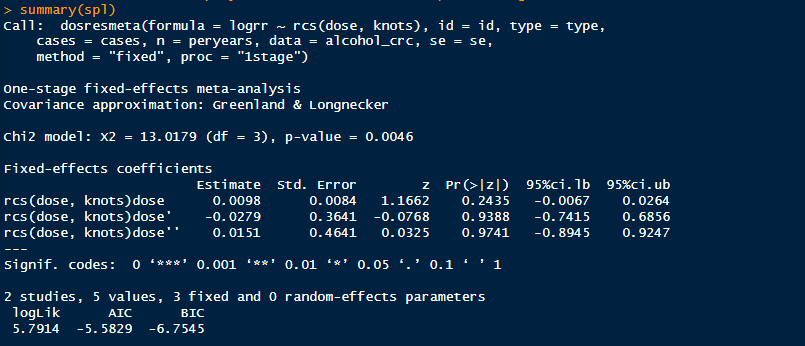

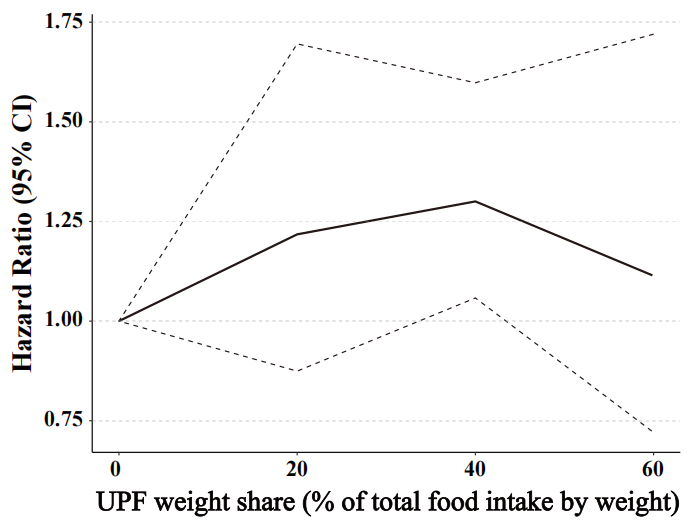


**UPF weight share (% of total food intake by weight)**


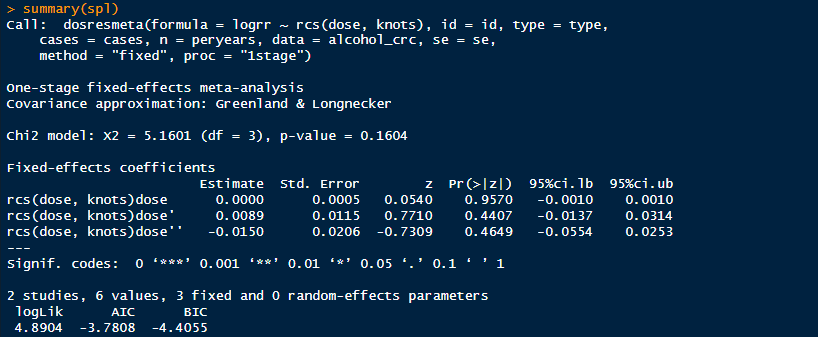

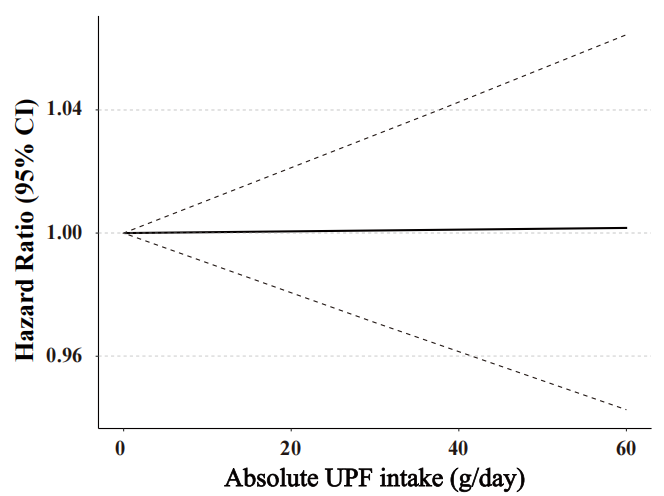


**Absolute UPF intake (g/day)**

To further assess whether a non-linear dose–response relationship exists between UPF intake and the risk of digestive system cancers, we conducted dose–response analyses using restricted cubic spline models. Given the heterogeneity in exposure metrics across the included studies, UPF intake was analysed separately using two exposure scales: the percentage contribution of UPF to total daily food intake by weight (UPF weight share, %g/day) and absolute UPF intake (g/day). UPF weight share reflects the relative contribution of UPF within the overall dietary structure, whereas g/day captures the absolute amount of UPF consumed. These two metrics characterise UPF exposure from different perspectives and were therefore modelled and interpreted separately.

In the primary dose–response analysis based on UPF weight share, the restricted cubic spline model suggested a potential non-linear association between the proportion of UPF intake and the risk of digestive system cancers. The linear dose–response analysis showed that each 10-percentage-point increment in UPF weight share was associated with a higher risk of digestive system cancers. Further testing for non-linearity yielded a statistically significant result (P for non-linearity = 0.0046), suggesting that the association may not follow a simple proportional linear increase. Overall, the non-linear curve showed an upward trend in the point estimates of cancer risk with increasing UPF weight share, with relatively higher risk estimates observed around an intake level of approximately 40%. These findings suggest that the association between UPF intake and digestive system cancer risk may become more apparent when UPF accounts for a relatively high proportion of the overall diet.

However, this non-linear pattern should be interpreted with caution. First, the number of studies available for the %g/day dose–response analysis was limited, and data support at both the lower and higher ends of the exposure distribution was relatively sparse. Consequently, the 95% confidence intervals became wider in these exposure ranges, with some intervals crossing the null value. Second, although the test for non-linearity was statistically significant, the curve did not demonstrate a clear, stable, or reproducible risk threshold, nor did it indicate an abrupt increase in risk above a specific level of UPF weight share. Therefore, this finding is more appropriately interpreted as evidence of a potential non-linear trend rather than as evidence of a definitive clinical or public health threshold. In other words, the current evidence supports an association between higher UPF weight share and increased risk of digestive system cancers, but it is insufficient to define a precise cut-off point for UPF intake.

In the supplementary dose–response analysis based on g/day, absolute UPF intake was generally positively associated with the risk of digestive system cancers. The linear model showed that each 100 g/day increment in UPF intake was associated with a modest increase in cancer risk. In contrast to the analysis based on UPF weight share, the test for non-linearity on the g/day scale was not statistically significant (P for non-linearity = 0.160), suggesting that, within the range of currently available data, the relationship between absolute UPF intake and digestive system cancer risk can be approximately interpreted as linear. That is, based on the available studies, increasing absolute UPF intake was associated with higher cancer risk, but no clear curvilinear pattern, plateau effect, or threshold effect was observed.

The differences observed between the two exposure scales may be related to their distinct epidemiological interpretations. UPF weight share reflects the proportion of UPF within the overall dietary pattern and may therefore better capture the extent to which unprocessed or minimally processed foods are displaced by UPF. In contrast, g/day reflects the absolute weight of UPF consumed and may be influenced by total food intake, energy intake, dietary assessment methods, and differences in water content across UPF items. Thus, the potential non-linear trend observed on the %g/day scale may suggest that the associated risk becomes more pronounced when UPF constitutes a larger proportion of the overall diet. By contrast, the approximately linear trend observed on the g/day scale suggests that greater absolute UPF intake itself may also be associated with increased risk, although without a clear non-linear pattern.

Overall, the dose–response analyses suggest an exposure-gradient relationship between increasing UPF intake and higher risk of digestive system cancers. The primary analysis based on UPF weight share supports a potential non-linear trend; however, given the limited number of included studies and the wide confidence intervals at extreme exposure levels, the curve should not be interpreted as indicating a definitive risk threshold. The supplementary analysis based on g/day showed an approximately linear association. Taken together, these findings support an association between higher UPF intake and increased risk of digestive system cancers. Nevertheless, further prospective studies using consistent UPF classifications, standardised intake metrics, repeated dietary assessments, and adequate reporting of dose distributions are needed to clarify the shape of this dose–response relationship.

# Supplementary Figure S1. Forest plot of the association between UPF intake and colorectal cancer risk in cohort studies.


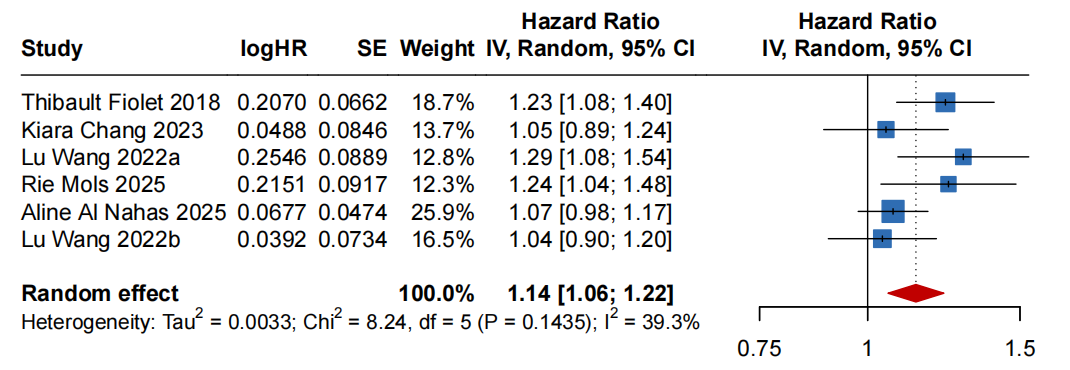


# Supplementary Figure S2. Forest plot of the association between UPF intake and colon cancer risk in cohort studies.


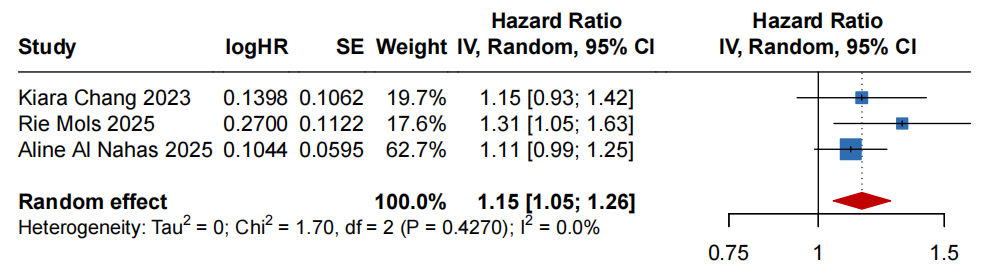


# Supplementary Figure S3. Forest plot of the association between UPF intake and proximal colon cancer risk in cohort studies.


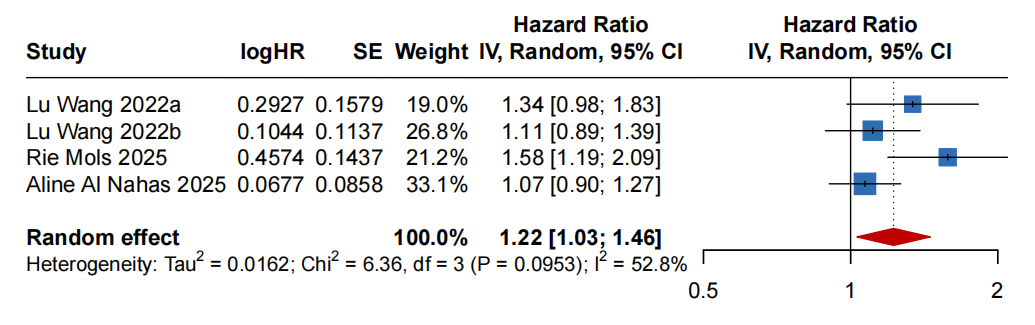


# Supplementary Figure S4. Forest plot of the association between UPF intake and distal colon cancer risk in cohort studies.


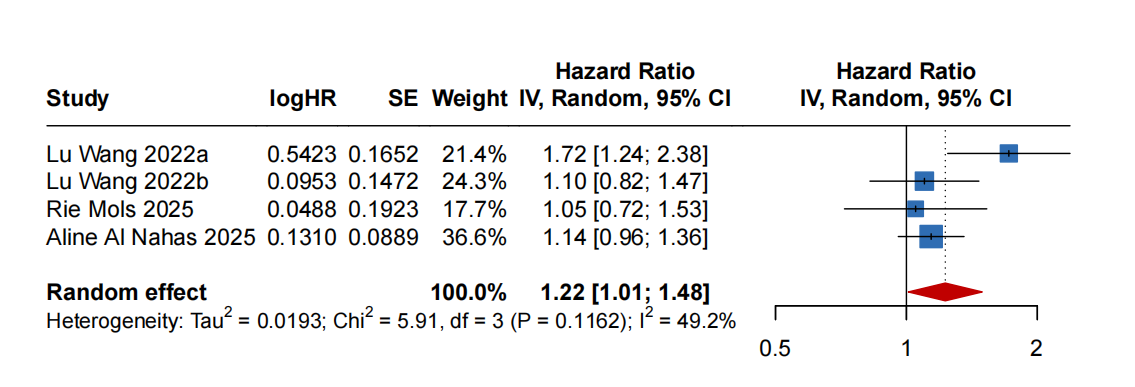


# Supplementary Figure S5. Forest plot of the association between UPF intake and rectal cancer risk in cohort studies.


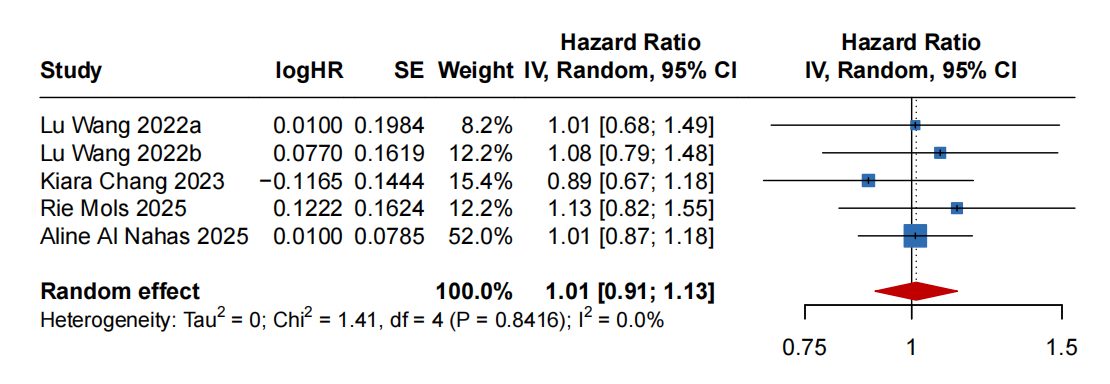


# Supplementary Figure S6. Forest plot of the association between UPF intake and oesophageal adenocarcinoma risk in cohort studies.


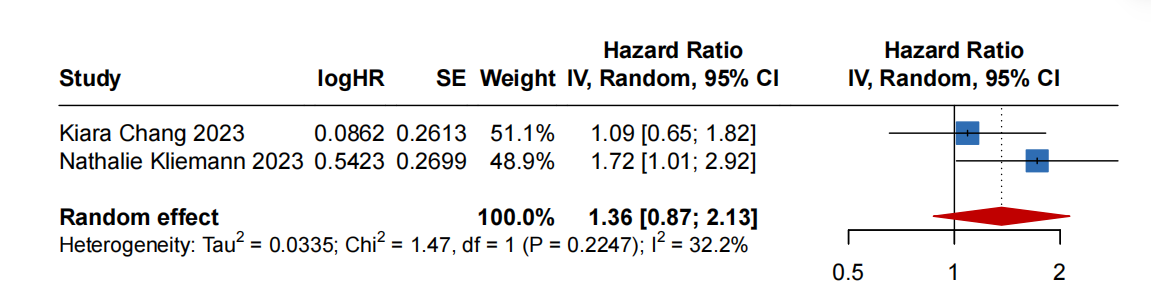


# Supplementary Figure S7. Forest plot of the association between UPF intake and oesophageal squamous cell carcinoma risk in cohort studies.


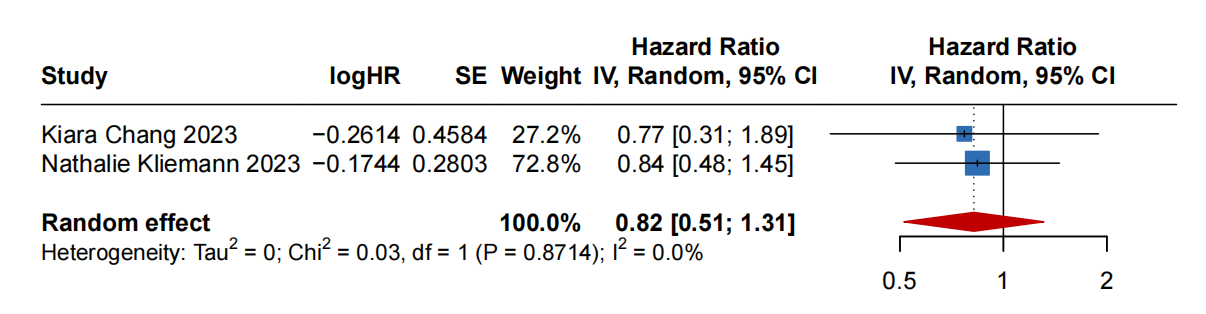


# Supplementary Figure S8. Forest plot of the association between UPF intake and gastric cardia cancer risk in cohort studies.


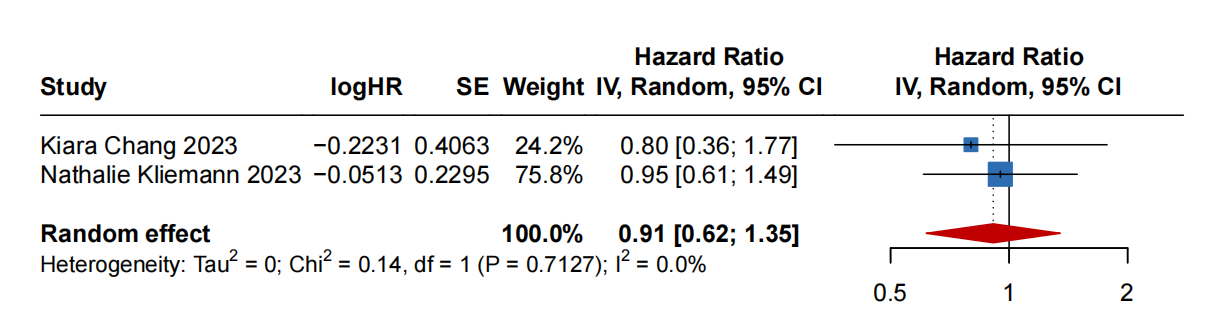


# Supplementary Figure S9. Forest plot of the association between UPF intake and non-cardia gastric cancer risk in cohort studies.


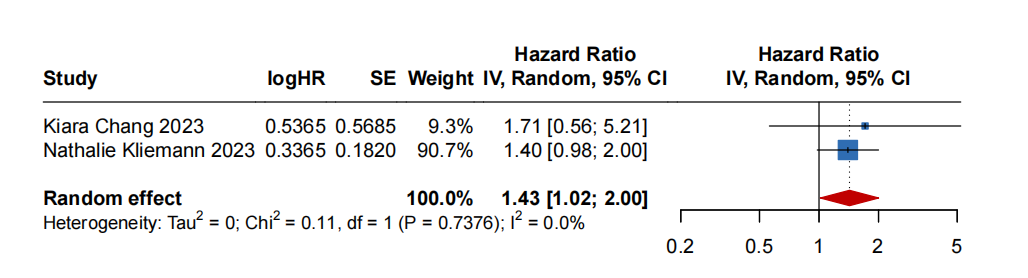


# Supplementary Figure S10. Forest plot of the association between UPF intake and liver cancer risk in cohort studies.


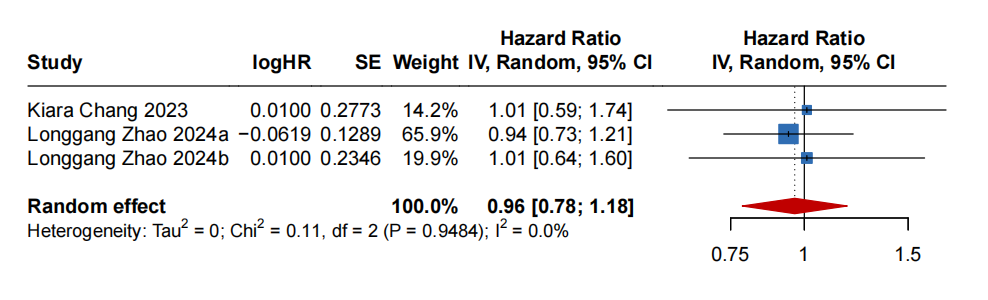


# Supplementary Figure S11. Forest plot of the association between UPF intake and hepatocellular carcinoma risk in cohort studies.


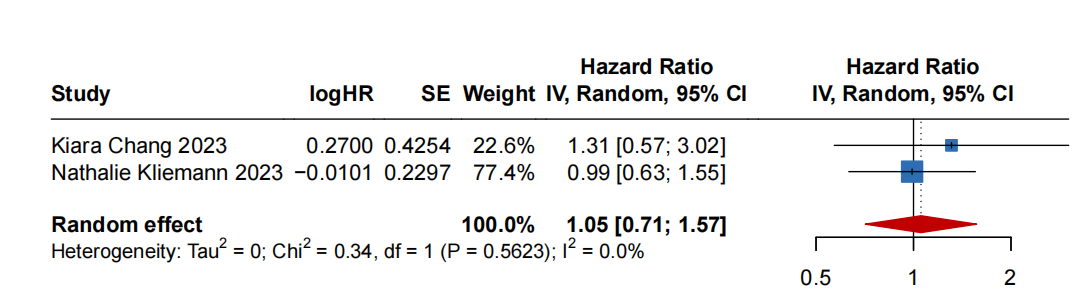


# Supplementary Figure S12. Forest plot of the association between UPF intake and pancreatic cancer risk in cohort studies.


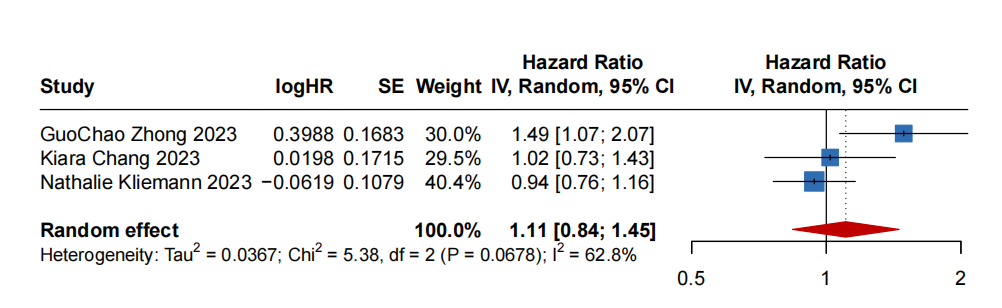


# Supplementary Figure S13. Forest plot of the association between UPF intake and colorectal cancer risk in case-control studies.


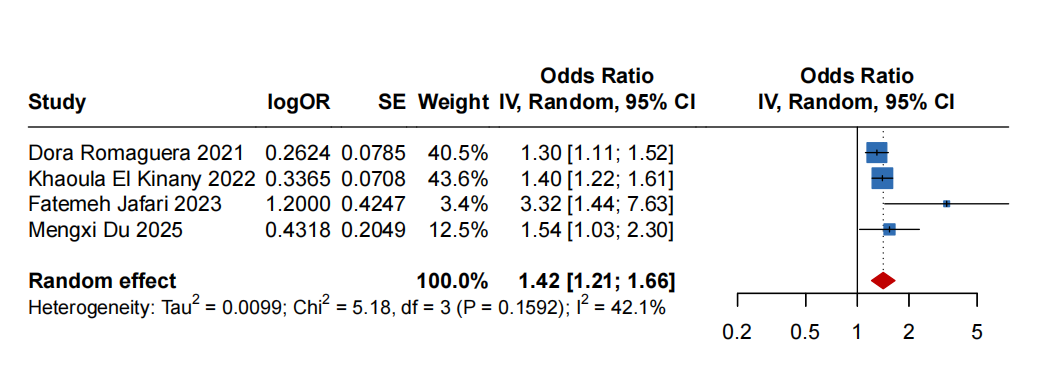


# Supplementary Figure S14. Forest plot of the association between UPF intake and colon cancer risk in case-control studies.


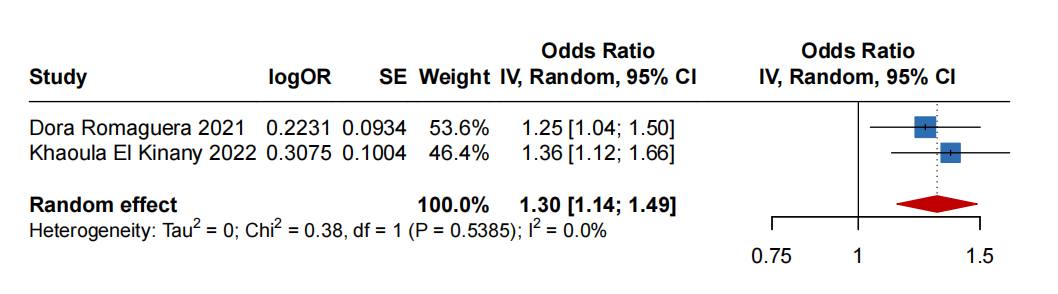


# Supplementary Figure S15. Forest plot of the association between UPF intake and rectal cancer risk in case-control studies.


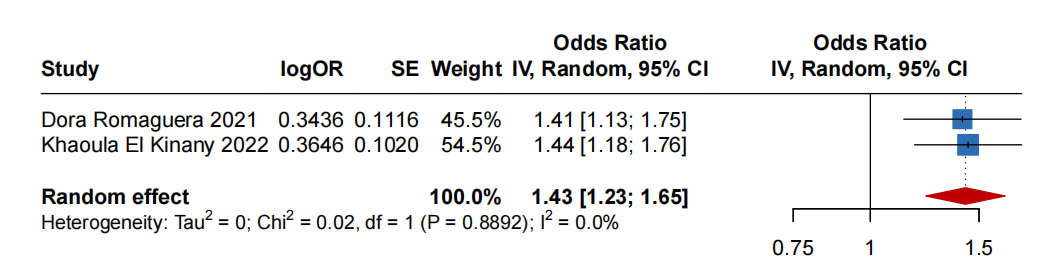


# Supplementary Figure S16. Forest plot of the association between UPF meat/protein product intake and digestive system cancer risk in cohort studies.


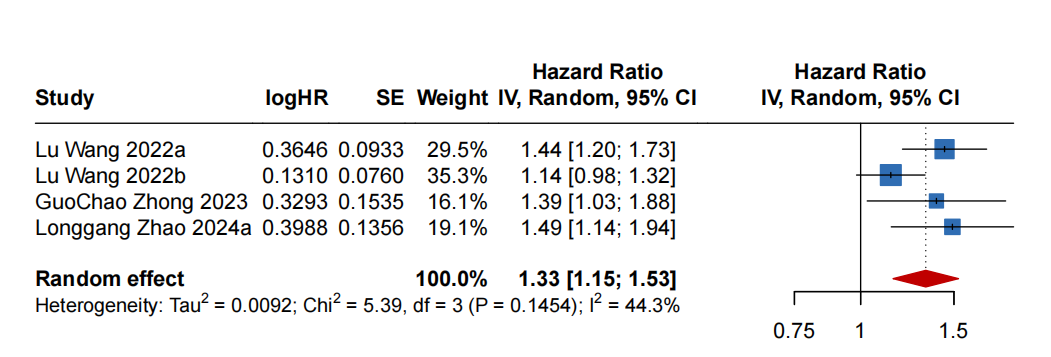


# Supplementary Figure S17. Forest plot of the association between UPF beverage intake and digestive system cancer risk in cohort studies.


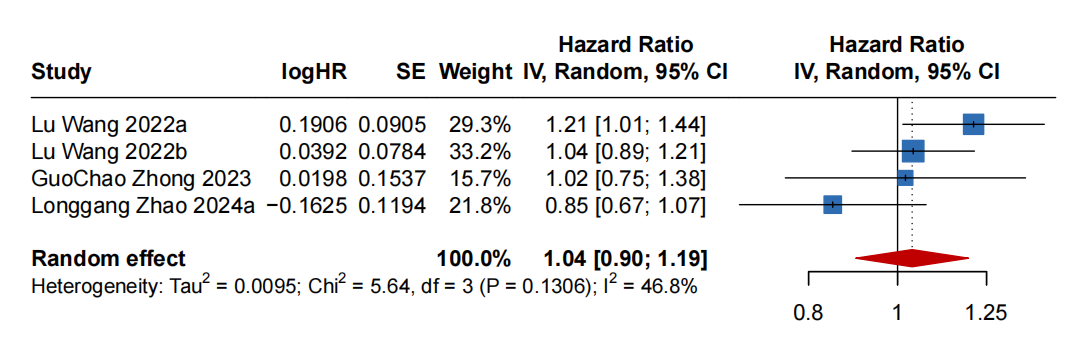


# Supplementary Figure S18. Forest plot of the association between UPF ready meal intake and digestive system cancer risk in cohort studies.


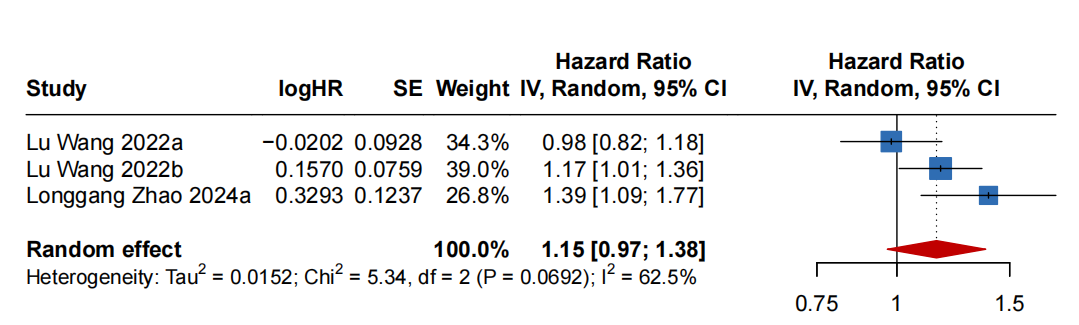


# Supplementary Figure S19. Forest plot of the association between UPF grain/bread product intake and digestive system cancer risk in cohort studies.


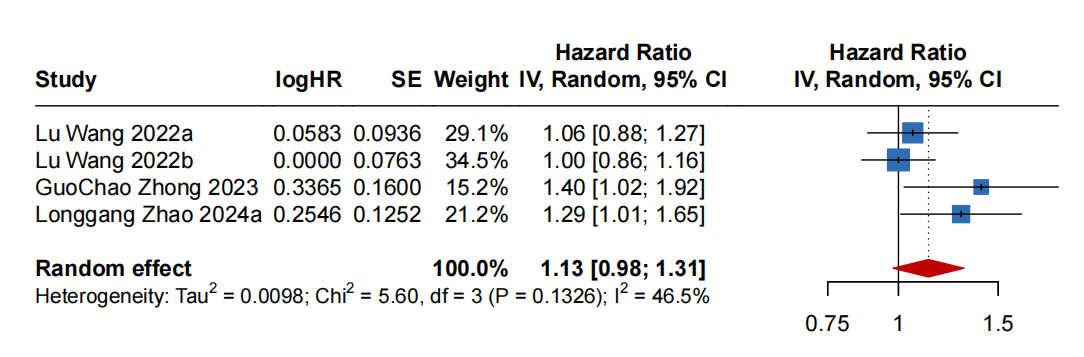


# Supplementary Figure S20. Forest plot of the association between UPF sweet snack/dessert intake and digestive system cancer risk in cohort studies.


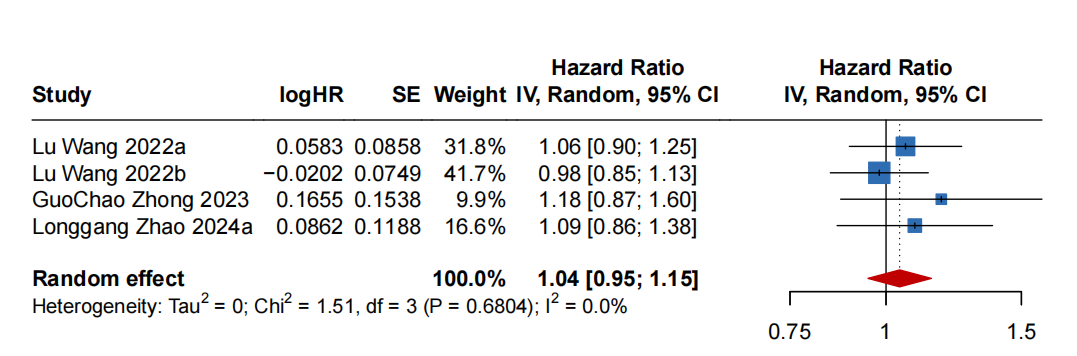


# Supplementary Figure S21. Meta-analysis of the association between UPF intake and digestive system cancer risk in cohort studies stratified by sex.


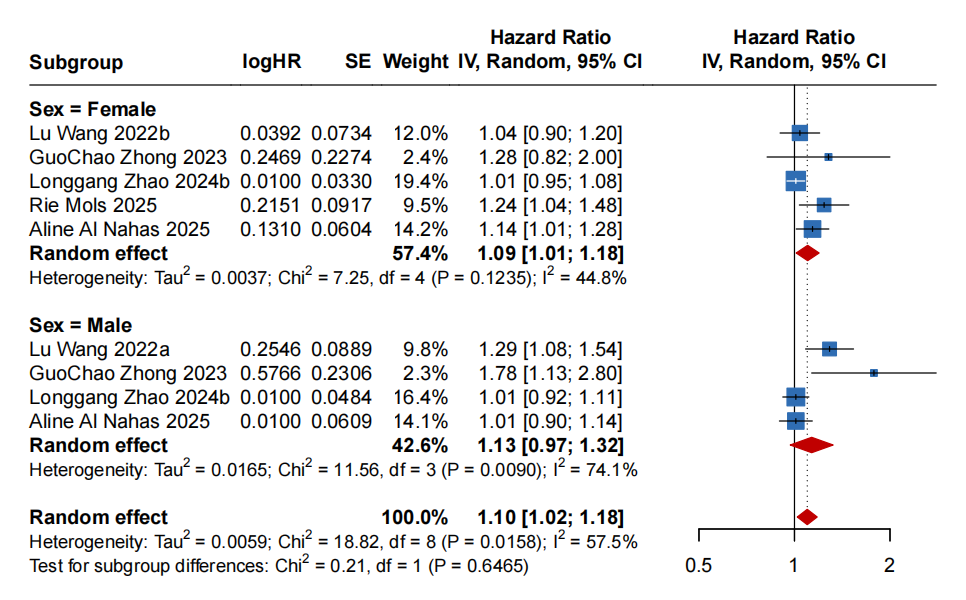


# Supplementary Figure S22. Meta-analysis of the association between UPF intake and digestive system cancer risk in cohort studies stratified by geographic region.


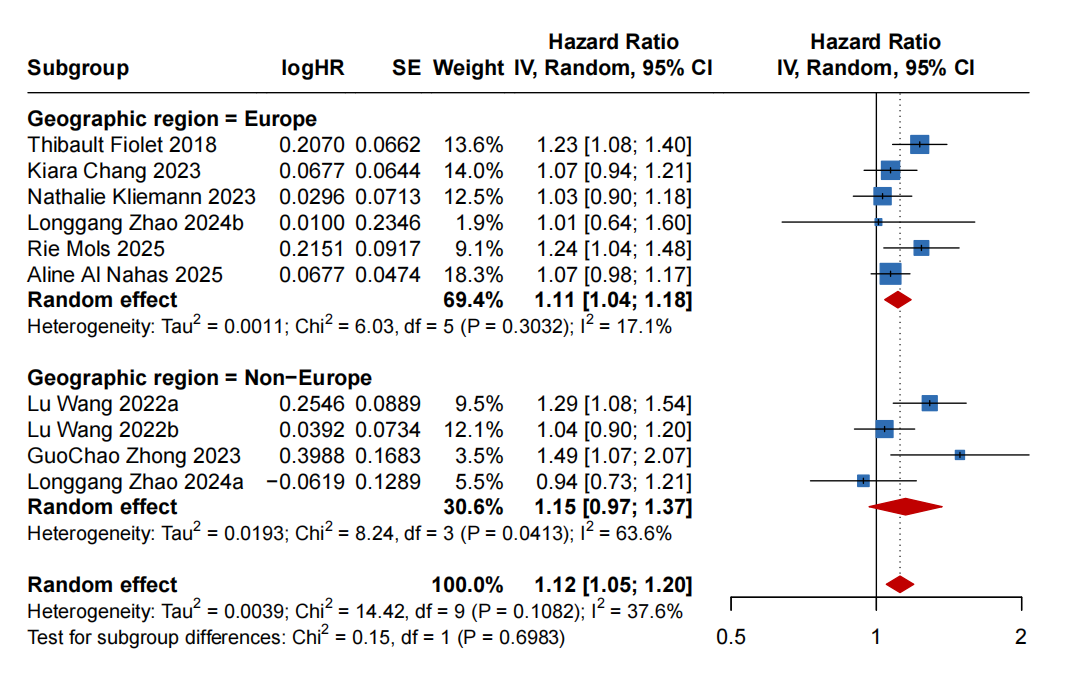


# Supplementary Figure S23. Meta-analysis of the association between UPF intake and digestive system cancer risk in cohort studies stratified by mean follow-up duration.


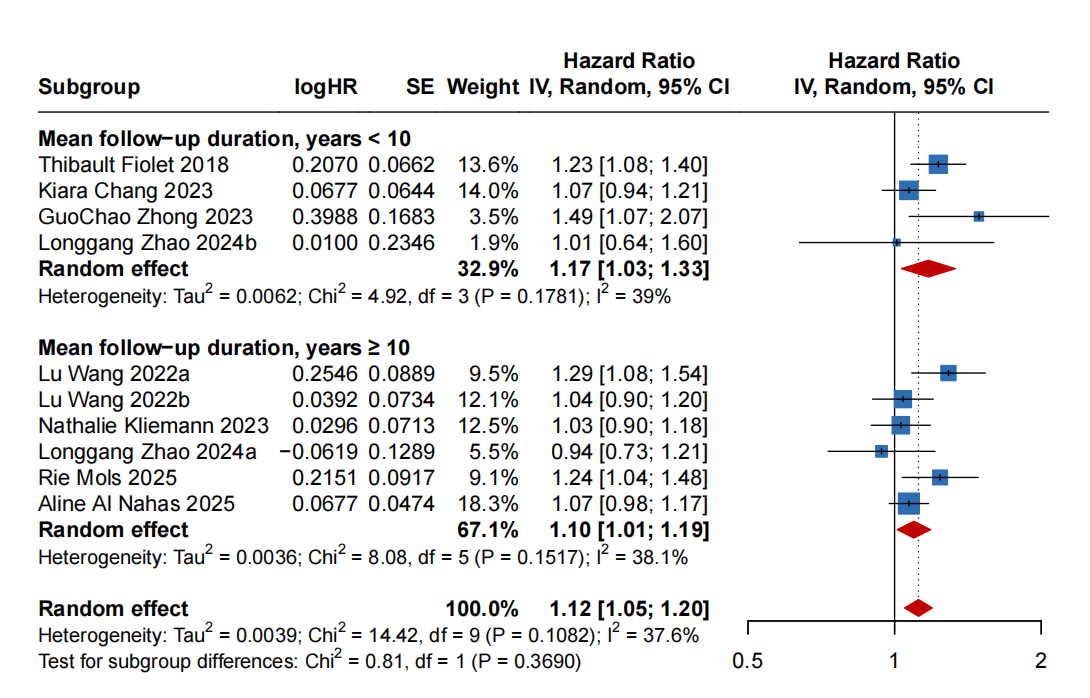


# Supplementary Figure S24. Meta-analysis of the association between UPF intake and digestive system cancer risk in cohort studies stratified by mean age at baseline.


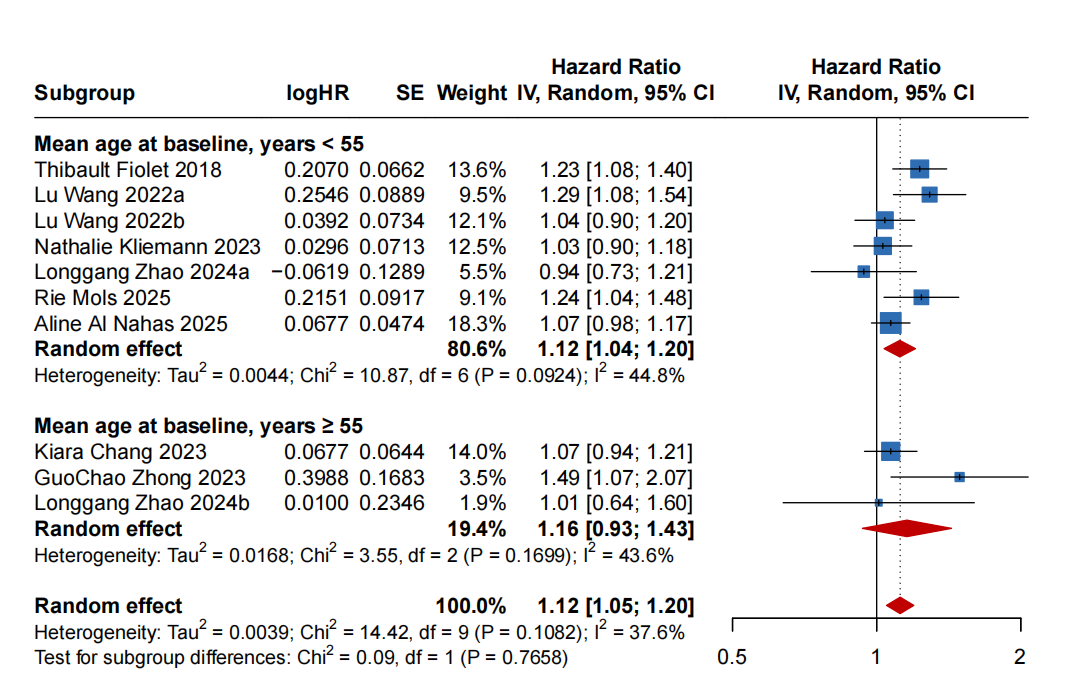


# Supplementary Figure S25. Meta-analysis of the association between UPF intake and digestive system cancer risk in cohort studies stratified by mean body mass index.


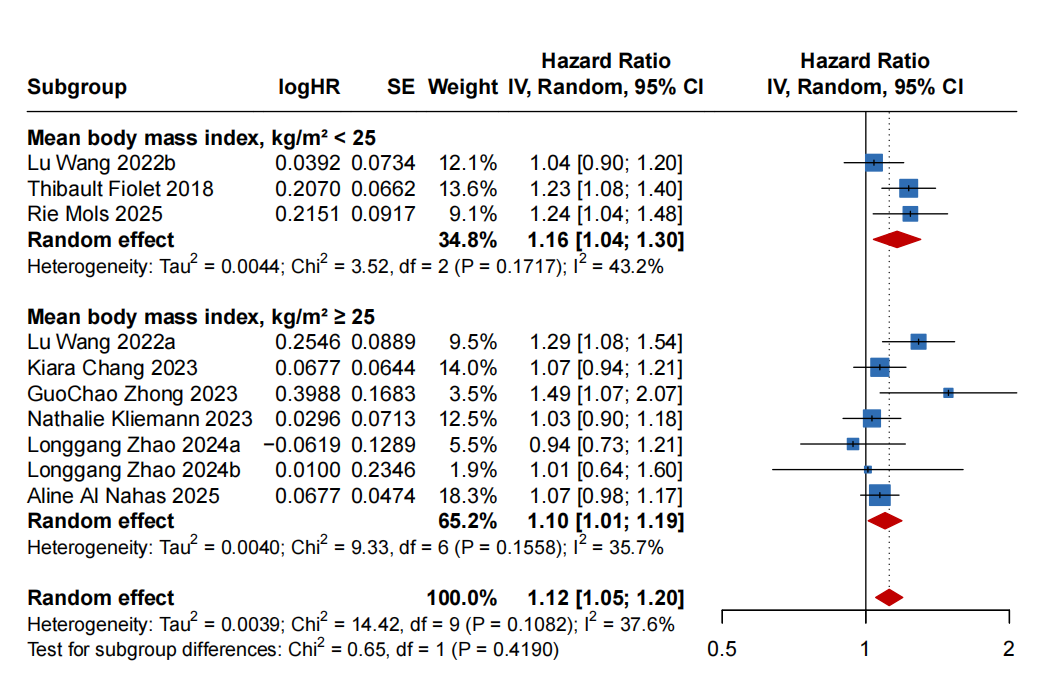


# Supplementary Figure S26. Meta-analysis of the association between UPF intake and digestive system cancer risk in cohort studies stratified by mean total energy intake.


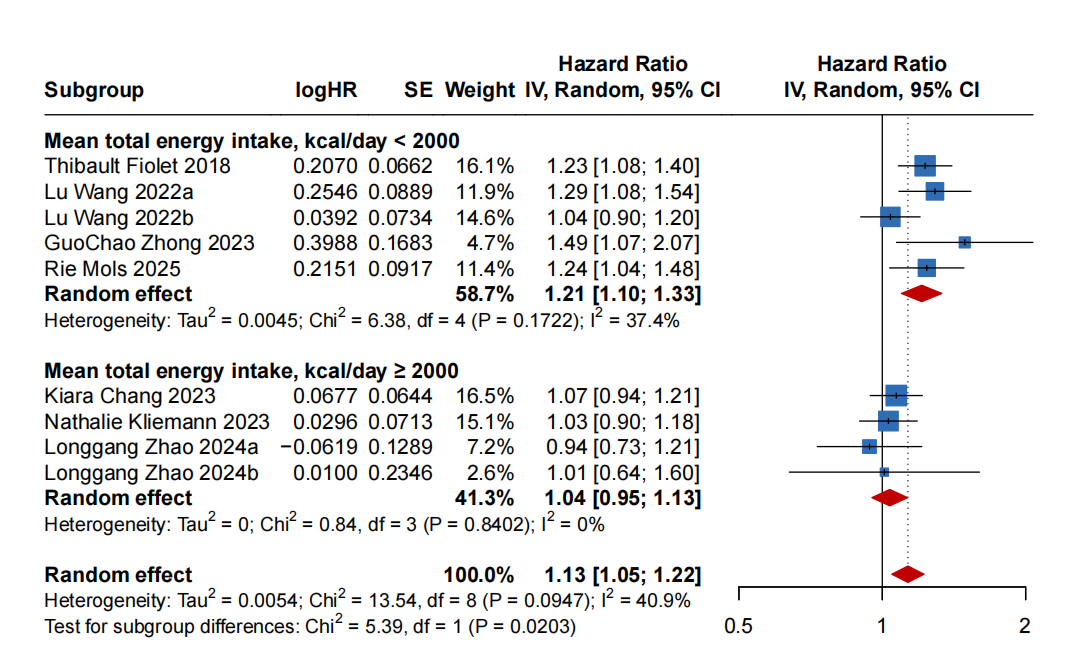


# Supplementary Figure S27. Meta-analysis of the association between UPF intake and digestive system cancer risk in cohort studies stratified by dietary assessment method.


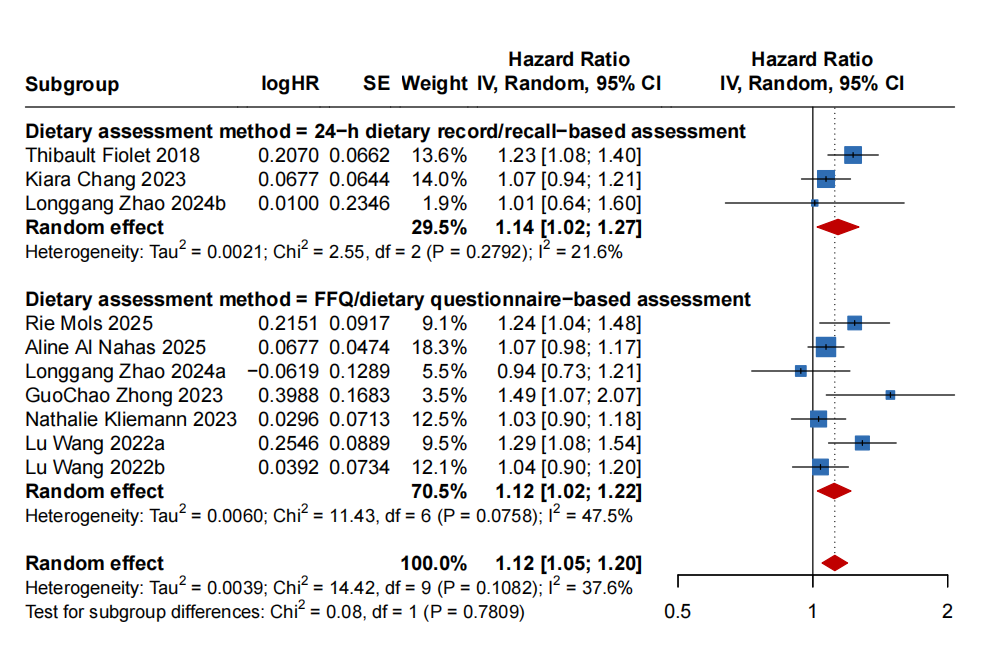


# Supplementary Figure S28. Meta-analysis of the association between UPF intake and digestive system cancer risk in case-control studies stratified by geographic region.


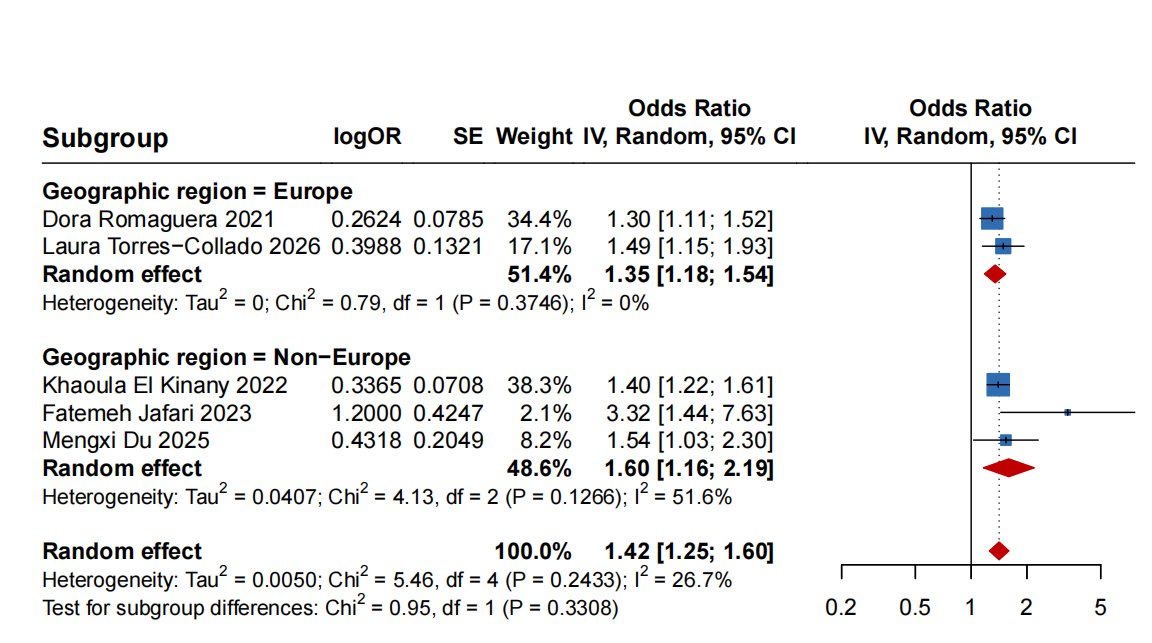


# Supplementary Figure S29. Meta-analysis of the association between UPF intake and digestive system cancer risk in case-control studies stratified by mean total energy intake.


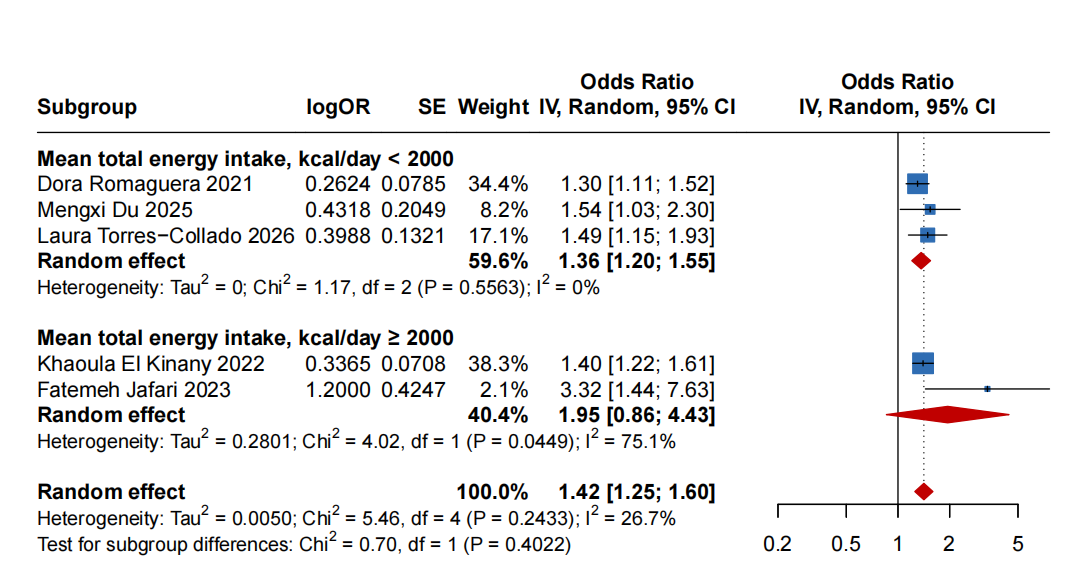


# Supplementary Figure S30. Meta-analysis of the association between UPF intake and digestive system cancer risk in case-control studies stratified by source of controls.


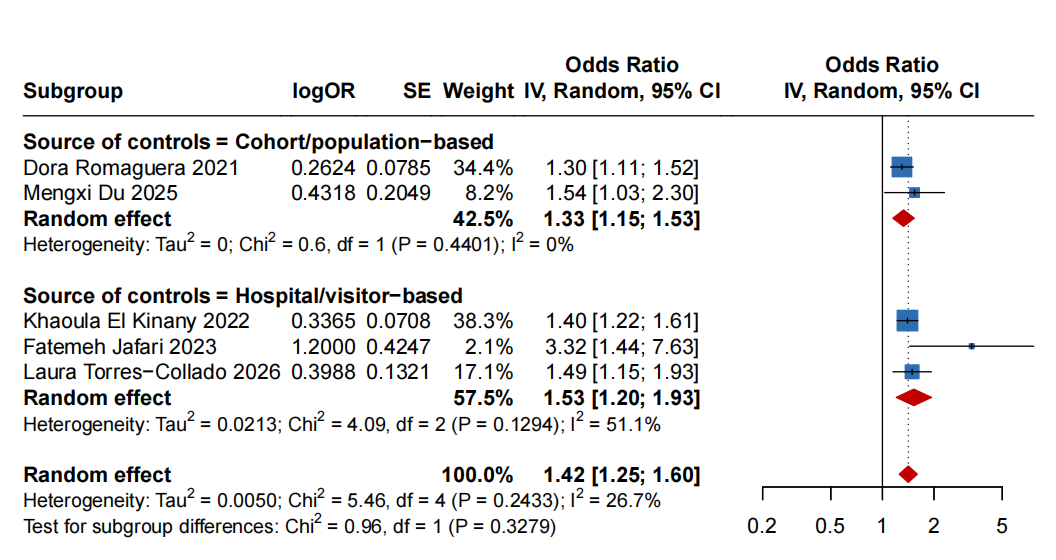


# Supplementary Figure S31. Forest plot of the association between each 10-percentage-point increase in UPF weight share and digestive system cancer risk in cohort studies.


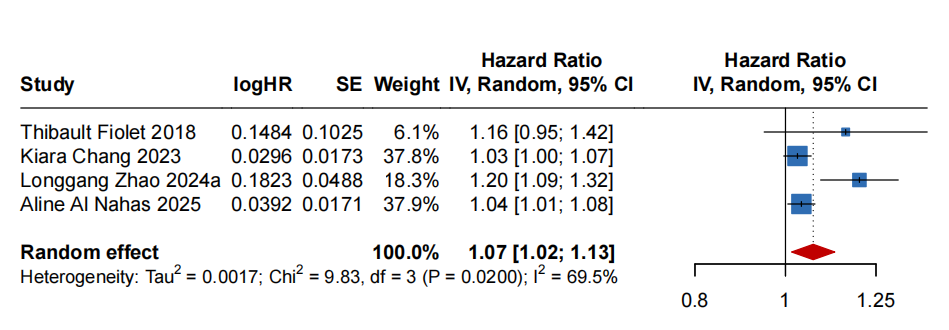


# Supplementary Figure S32. Forest plot of study-specific summary estimates for the dose–response association between UPF weight share (%g/day) and digestive system cancer risk in cohort studies.


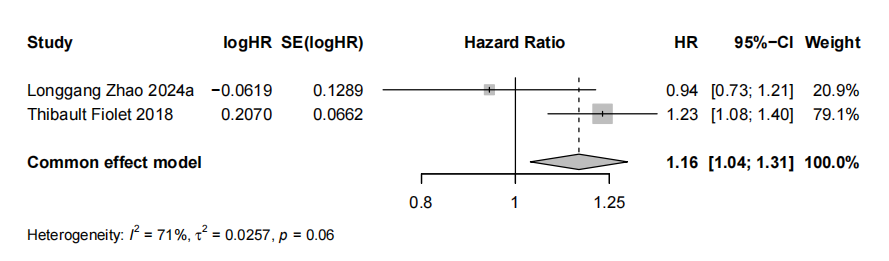


# Supplementary Figure S33. Forest plot of study-specific summary estimates for the dose–response association between absolute UPF intake (g/day) and digestive system cancer risk in cohort studies.


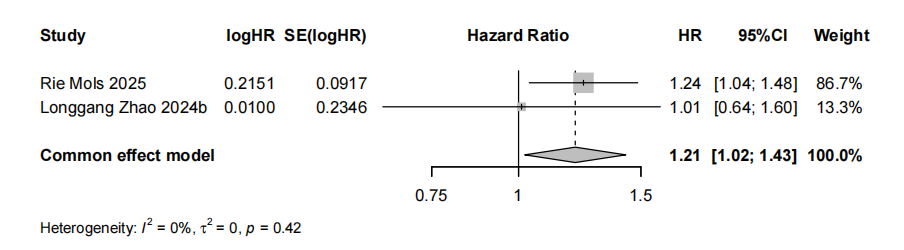


# Supplementary Figure S34. Funnel plot and Egger’s test for publication bias in the association between UPF intake and digestive system cancer risk in cohort studies.

# Supplementary Figure S35. Funnel plot and Egger’s test for publication bias in the association between UPF intake and digestive system cancer risk in case-control studies.

# Supplementary Figure S36. Leave-one-out sensitivity analysis of the association between UPF intake and digestive system cancer risk in cohort studies.

# Supplementary Figure S37. Leave-one-out sensitivity analysis of the association between UPF intake and digestive system cancer risk in case-control studies.

# Supplementary Figure S38. Leave-one-out sensitivity analysis of the association between UPF meat/protein product intake and digestive system cancer risk in cohort studies.

# Supplementary Table S1. The treatment of duplicate data.

In this meta-analysis, we performed a thorough and systematic screening of overlapping data sources and exposure indicators to ensure the independence and reliability of the 14 included studies. For these six studies with overlapping subject populations, we applied the following strict exclusion strategy: ultimately, four studies reporting outcomes for different diseases were included in the meta-analysis: (1) Exclusion of studies with complete temporal overlap: when the time span of one study completely overlapped with another, and the results reported by both studies were consistent, we decisively excluded the overlapped study to prevent data duplication and bias; (2) Handling of partial results: if the time span of one study overlapped with another, but the latter reported results not covered by the former, we carefully excluded the overlapping data from the meta-analysis to ensure the independence and completeness of the results; (3) Selection of studies with partial temporal overlap: for studies with partially overlapping time spans, we prioritized retaining those with larger sample sizes or longer study periods, and excluded studies with smaller sample sizes or shorter durations to enhance the representativeness and statistical validity of the data.

**eTable 1. The treatment of duplicate data**

| **Study** | **Data sources** | **Study period** | **Study population** | **Exposure assessment** |
| --- | --- | --- | --- | --- |
| ^1^Nathalie Kliemann 2023 | EPIC, European Prospective Investigation into Cancer and Nutrition | 1991-2013 | General population | colorectal cancer, colon cancer, proximal colon cancer, distal colon cancer, rectal cancer, oesophageal adenocarcinoma, oesophageal squamous cell carcinoma, gastric cardia cancer, gastric non-cardia cancer  hepatocellular carcinoma, gallbladder cancer, pancreatic cancer |
| ^2^Fernanda Morales-Berstein 2024* | EPIC, European Prospective Investigation into Cancer and Nutrition | 1991-2013 | General population | oesophageal adenocarcinoma |
| ^3^Aline Al Nahas 2025 | EPIC, European Prospective Investigation into Cancer and Nutrition | 1992-2013 | General population | colorectal cancer, colon cancer, proximal colon cancer, distal colon cancer, rectal cancer |
| ^4^Jeroen Berden 2026* | EPIC, European Prospective Investigation into Cancer and Nutrition | 1992-2013 | General population | colorectal cancer, colon cancer, rectal cancer |
| ^5^Kiara Chang 2023 | UK Biobank | 2007-2021 | General population | gastrointestinal cancer, oesophagus cancer, oesophageal adenocarcinoma, oesophageal squamous cell carcinoma, stomach cancer, stomach cardia cancer, stomach non-cardia cancer, small intestine cancer, colorectal cancer, colon cancer rectum cancer, anal cancer, hepatobiliary tract cancer, liver cancer, hepatocellular carcinoma, intrahepatic bile duct cancer, pancreatic cancer |
| ^6^Longgang Zhao 2024b | UK Biobank | 2006-2020 | General population | liver cancer |

* Studies marked with an asterisk were excluded from the present meta-analysis because of duplicate or overlapping cohort data.

Several studies were derived from the EPIC cohort. Jeroen Berden et al. (2026) reported colorectal cancer, colon cancer, and rectal cancer outcomes based on EPIC data from 1992 to 2013. Because Aline Al Nahas et al. (2025) used a largely overlapping EPIC cohort period, included a larger analytical population, and provided more detailed colorectal cancer site-specific estimates, including proximal colon cancer and distal colon cancer, the study by Berden et al. was excluded to avoid duplicate use of EPIC colorectal cancer data. Fernanda Morales-Berstein et al. (2024) also used the EPIC cohort and reported oesophageal adenocarcinoma. This outcome overlapped with that reported in the broader multi-cancer EPIC analysis by Nathalie Kliemann et al. (2023), which included oesophageal adenocarcinoma together with other digestive system cancer outcomes. Therefore, Morales-Berstein et al. was excluded from the quantitative synthesis because the same EPIC-based oesophageal adenocarcinoma estimate was already represented by Kliemann et al. The study by Kliemann et al. was retained for digestive cancer outcomes not otherwise represented by more detailed EPIC reports. However, to avoid duplication with the colorectal cancer-specific EPIC analysis by Al Nahas et al., colorectal cancer, colon cancer, and rectal cancer estimates from Kliemann et al. were not included in the corresponding colorectal site-specific pooled analyses. The study by Al Nahas et al. was retained for colorectal cancer-related outcomes because it provided more detailed and outcome-specific estimates for colorectal cancer, colon cancer, proximal colon cancer, distal colon cancer, and rectal cancer.

For the UK Biobank cohort, both Kiara Chang et al. (2023) and Longgang Zhao et al. (2024b) reported liver cancer-related outcomes. However, the two studies used different UPF exposure metrics: Chang et al. reported UPF intake as the percentage of total food intake by weight (%g/day), whereas Zhao et al. reported energy-adjusted UPF intake in g/day. Therefore, these studies were used only in analyses corresponding to their respective exposure metrics and were not entered simultaneously into the same pooled estimate for an identical exposure–outcome comparison.

**References:**

[1] Kliemann N, Rauber F, Bertazzi Levy R, et al. Food processing and cancer risk in Europe: results from the prospective EPIC cohort study. Lancet Planet Health. 2023;7(3):e219-e232. doi:10.1016/S2542-5196(23)00021-9 .

[2] Morales-Berstein F, Biessy C, Viallon V, et al. Ultra-processed foods, adiposity and risk of head and neck cancer and oesophageal adenocarcinoma in the European Prospective Investigation into Cancer and Nutrition study: a mediation analysis. *Eur J Nutr*. 2024;63(2):377-396. doi:10.1007/s00394-023-03270-1.

[3] Al Nahas A, Yammine Ghantous S, Morales Berstein F, et al. Associations between degree of food processing and colorectal cancer risk in a large-scale European cohort. *Int J Cancer*. 2025;157(2):260-276. doi:10.1002/ijc.35361.

[4] Berden J, Langselius O, González-Gil EM, et al. Assessing the role of ultra-processed foods in colorectal cancer incidence: insights from the EPIC cohort. *BMC Public Health*. Published online April 22, 2026. doi:10.1186/s12889-026-27225-3.

[5] Chang K, Gunter MJ, Rauber F, et al. Ultra-processed food consumption, cancer risk and cancer mortality: a large-scale prospective analysis within the UK Biobank. *EClinicalMedicine*. 2023;56:101840. Published 2023 Jan 31. doi:10.1016/j.eclinm.2023.101840.

[6] Zhao L, Clay-Gilmour A, Zhang J, Zhang X, Steck SE. Higher ultra-processed food intake is associated with adverse liver outcomes: a prospective cohort study of UK Biobank participants. *Am J Clin Nutr*. 2024;119(1):49-57. doi:10.1016/j.ajcnut.2023.10.014.

# Supplementary Table S2. Summary of meta-analysis results for the association between UPF intake and digestive system cancer risk.

| **Cancer outcome** | **No. of studies** | **Pooled estimate (95% CI)** | **Tau²** | **Test for heterogeneity** | |
| --- | --- | --- | --- | --- | --- |
|  |  |  |  | **I^2^ (%)** | ***p*-value** |
| **Study design:** **Cohort studies** | | | | | |
| Overall digestive system cancers | 9 | HR 1.12 (1.05–1.20) | 0.0039 | 37.6 | 0.1082 |
| Colorectal cancer | 5 | HR 1.14 (1.06–1.22) | 0.0033 | 39.3 | 0.1435 |
| Colon cancer | 3 | HR 1.15 (1.05–1.26) | 0 | 0.0 | 0.4270 |
| Proximal colon cancer | 3 | HR 1.22 (1.03–1.46) | 0.0162 | 52.8 | 0.0953 |
| Distal colon cancer | 3 | HR 1.22 (1.01–1.48) | 0.0193 | 49.2 | 0.1162 |
| Rectal cancer | 4 | HR 1.01 (0.91–1.13) | 0 | 0.0 | 0.8416 |
| Oesophageal adenocarcinoma | 2 | HR 1.36 (0.87–2.13) | 0.0335 | 32.2 | 0.2247 |
| Oesophageal squamous cell carcinoma | 2 | HR 0.82 (0.51–1.31) | 0 | 0.0 | 0.8714 |
| Gastric cardia cancer | 2 | HR 0.91 (0.62–1.35) | 0 | 0.0 | 0.7127 |
| Non-cardia gastric cancer | 2 | HR 1.43 (1.02–2.00) | 0 | 0.0 | 0.7376 |
| Liver cancer | 3 | HR 0.96 (0.78–1.18) | 0 | 0.0 | 0.9484 |
| Hepatocellular carcinoma | 2 | HR 1.05 (0.71–1.57) | 0 | 0.0 | 0.5623 |
| Pancreatic cancer | 3 | HR 1.11 (0.84–1.45) | 0.0367 | 62.8 | 0.0678 |
| **Study design:** **Case-control studies** | | | | | |
| Overall digestive system cancers | 5 | OR 1.42 (1.25–1.60) | 0.0050 | 26.7 | 0.2433 |
| Colorectal cancer | 4 | OR 1.42 (1.21–1.66) | 0.0099 | 42.1 | 0.1592 |
| Colon cancer | 2 | OR 1.30 (1.14–1.49) | 0 | 0.0 | 0.5385 |
| Rectal cancer | 2 | OR 1.43 (1.23–1.65) | 0 | 0.0 | 0.8892 |

Note. Pooled hazard ratios (HRs) are reported for cohort studies and pooled odds ratios (ORs) for case-control studies. Estimates compare the highest versus the lowest category of ultra-processed food or ultra-processed dietary intake and were derived using inverse-variance random-effects models. P values refer to Cochran's Q test for heterogeneity. CI, confidence interval; UPF, ultra-processed food.

# Supplementary Table S3. Literature was excluded after full-text review for failing to meet the inclusion criteria.

| **Study year** | **References** | **Reason** |
| --- | --- | --- |
| Omonefe O Omofuma 2020 | 1. Omofuma OO, Turner DP, Peterson LL, Merchant AT, Zhang J, Steck SE. Dietary Advanced Glycation End-products (AGE) and Risk of Breast Cancer in the Prostate, Lung, Colorectal and Ovarian Cancer Screening Trial (PLCO). *Cancer Prev Res (Phila)*. 2020;13(7):601-610. doi:10.1158/1940-6207.CAPR-19-0457. | Irrelevant study design. |
| Jana Sremanakova 2024 | 1. Sremanakova J, Sowerbutts AM, Todd C, et al. Healthy Eating and Active Lifestyle after Bowel Cancer (HEAL ABC)-feasibility randomised controlled trial. *Eur J Clin Nutr*. 2024;78(12):1095-1104. doi:10.1038/s41430-024-01491-z. | Irrelevant study design. |
| Mengxi Du 2025 | 1. Du M, Wang X, Hang D, et al. Metabolomic pattern of ultraprocessed food intake and its association with colorectal cancer risk. *Gut*. Published online December 24, 2025. doi:10.1136/gutjnl-2025-335618. | Irrelevant study design. |
| Naomi Fliss-Isakov 2020 | 1. Fliss-Isakov N, Zelber-Sagi S, Ivancovsky-Wajcman D, Shibolet O, Kariv R. Ultra-Processed Food Intake and Smoking Interact in Relation with Colorectal Adenomas. *Nutrients*. 2020;12(11):3507. Published 2020 Nov 14. doi:10.3390/nu12113507. | Irrelevant outcome. |
| GuoChao Zhong 2021 | 1. Zhong GC, Gu HT, Peng Y, et al. Association of ultra-processed food consumption with cardiovascular mortality in the US population: long-term results from a large prospective multicenter study. *Int J Behav Nutr Phys Act*. 2021;18(1):21. Published 2021 Feb 3. doi:10.1186/s12966-021-01081-3. | Irrelevant outcome. |
| JianYuan Pu 2023 | 1. Pu JY, Xu W, Zhu Q, et al. Prediagnosis ultra-processed food consumption and prognosis of patients with colorectal, lung, prostate, or breast cancer: a large prospective multicenter study. *Front Nutr*. 2023;10:1258242. Published 2023 Oct 2. doi:10.3389/fnut.2023.1258242. | Irrelevant outcome. |
| Dong Hang 2023 | 1. Hang D, Wang L, Fang Z, et al. Ultra-processed food consumption and risk of colorectal cancer precursors: results from 3 prospective cohorts. J Natl Cancer Inst. 2023;115(2):155-164. doi:10.1093/jnci/djac221. | Irrelevant outcome. |
| Dong Hang 2024 | 1. Hang D, Du M, Wang L, et al. Ultra-processed food consumption and mortality among patients with stages I-III colorectal cancer: a prospective cohort study. *EClinicalMedicine*. 2024;71:102572. Published 2024 Mar 28. doi:10.1016/j.eclinm.2024.102572. | Irrelevant outcome. |
| Chen Wang 2026 | 1. Wang C, Du M, Kim H, et al. Ultraprocessed Food Consumption and Risk of Early-Onset Colorectal Cancer Precursors Among Women. *JAMA Oncol*. 2026;12(1):49-57. doi:10.1001/jamaoncol.2025.4777. | Irrelevant outcome. |
| Xinyu Wang 2026 | 1. Wang X, Du M, Hang D, et al. Metabolomic Pattern of Ultraprocessed Food Intake and Risk of Colorectal Cancer Precursors. *Gastroenterology*. Published online March 30, 2026. doi:10.1053/j.gastro.2026.02.044. | Irrelevant outcome. |
| Anthony Kityo 2026 | 1. Kityo A, Lee SA. Serum metabolites related to ultra-processed food intake in association with mortality: A prospective cohort study in the UK biobank. *Cancer Epidemiol*. Published online May 23, 2026. doi:10.1016/j.canep.2026.103114. | Irrelevant outcome. |
| Qingkun Song 2012 | 1. Song Q, Wang X, Yu IT, et al. Processed food consumption and risk of esophageal squamous cell carcinoma: A case-control study in a high risk area. *Cancer Sci*. 2012;103(11):2007-2011. doi:10.1111/j.1349-7006.2012.02387.x. | Irrelevant exposure. |
| SiHao Lin 2014 | 1. Lin SH, Li YH, Leung K, Huang CY, Wang XR. Salt processed food and gastric cancer in a Chinese population. *Asian Pac J Cancer Prev*. 2014;15(13):5293-5298. doi:10.7314/apjcp.2014.15.13.5293. | Irrelevant exposure. |
| Xudong Liu 2017 | 1. Liu X, Wang X, Lin S, et al. Dietary patterns and the risk of esophageal squamous cell carcinoma: A population-based case-control study in a rural population. *Clin Nutr*. 2017;36(1):260-266. doi:10.1016/j.clnu.2015.11.009. | Irrelevant exposure. |
| Rup Kumar Phukan 2018 | 1. Phukan RK, Borkakoty BJ, Phukan SK, et al. Association of processed food, synergistic effect of alcohol and HBV with Hepatocellular Carcinoma in a high incidence region of India. *Cancer Epidemiol*. 2018;53:35-41. doi:10.1016/j.canep.2018.01.005. | Irrelevant exposure. |
| Leolin Katsidzira 2018 | 1. Katsidzira L, Laubscher R, Gangaidzo IT, et al. Dietary patterns and colorectal cancer risk in Zimbabwe: A population based case-control study. *Cancer Epidemiol*. 2018;57:33-38. doi:10.1016/j.canep.2018.09.005. | Irrelevant exposure. |
| Marie Beslay 2021 | 1. Beslay M, Srour B, Deschasaux M, et al. Anxiety is a potential effect modifier of the association between red and processed meat consumption and cancer risk: findings from the NutriNet-Santé cohort. *Eur J Nutr*. 2021;60(4):1887-1896. doi:10.1007/s00394-020-02381-3. | Irrelevant exposure. |
| Monireh Sadat Seyyedsalehi 2022 | 1. Seyyedsalehi MS, Collatuzzo G, Rashidian H, et al. Dietary Ruminant and Industrial Trans-Fatty Acids Intake and Colorectal Cancer Risk. *Nutrients*. 2022;14(22):4912. Published 2022 Nov 20. doi:10.3390/nu14224912. | Irrelevant exposure. |
| Stela V Peres 2022 | 1. Peres SV, Silva DRM, Coimbra FJF, et al. Consumption of processed and ultra-processed foods by patients with stomach adenocarcinoma: a multicentric case-control study in the Amazon and southeast regions of Brazil. *Cancer Causes Control*. 2022;33(6):889-898. doi:10.1007/s10552-022-01567-w. | Irrelevant exposure. |
| Sook Yee Lim 2024 | 1. Lim SY, Ulaganathan V, Nallamuthu P, Gunasekaran B, Salvamani S. Dietary Patterns and Lifestyle Factors Associated with the Risk of Colorectal Cancer: A Hospital-Based Case-Control Study among Malaysians. *Malays J Med Sci*. 2024;31(1):212-234. doi:10.21315/mjms2024.31.1.18. | Irrelevant exposure. |
| Abdulbari Bener 2024 | 1. Bener A, Öztürk AE, Dasdelen MF, et al. Colorectal cancer and associated genetic, lifestyle, cigarette, nargileh-hookah use and alcohol consumption risk factors: a comprehensive case-control study. Oncol Rev. 2024;18:1449709. Published 2024 Oct 11. doi:10.3389/or.2024.1449709. | Irrelevant exposure. |
| Esther M González-Gil 2025 | 1. González-Gil EM, Matta M, Morales Berstein F, et al. Associations between degree of food processing and all-cause and cause-specific mortality: a multicentre prospective cohort analysis in 9 European countries. *Lancet Reg Health Eur*. 2025;50:101208. Published 2025 Jan 8. doi:10.1016/j.lanepe.2024.101208. | Irrelevant exposure. |
| Edgar Asiimwe 2025 | 1. Asiimwe E, Tolstykh I, Chan JM, et al. Adherence to World Cancer Research Fund/American Institute for Cancer Research Guidelines and Mortality among Participants with Colorectal Cancer in the MEC. *Cancer Epidemiol Biomarkers Prev*. 2025;34(9):1558-1565. doi:10.1158/1055-9965.EPI-25-0379. | Irrelevant exposure. |
| Longgang Zhao 2025 | 1. Zhao L, Chen Y, Clay-Gilmour A, Zhang J, Zhang X, Steck SE. Metabolomic and Proteomic Signatures of Ultra-processed Foods Are Positively Associated with Adverse Liver Outcomes. J Nutr. 2025;155(6):1851-1858. doi:10.1016/j.tjnut.2025.04.034. | Irrelevant exposure. |
| Emine Koc Cakmak 2025 | 1. Koc Cakmak E, Al Nahas A, Chimera B, et al. The 3V score and joint associations of low ultra-processed food, biodiverse and plant-based diets on colorectal cancer risk: results from the European Prospective Investigation into Cancer and Nutrition (EPIC) study. *EClinicalMedicine*. 2025;90:103662. Published 2025 Dec 1. doi:10.1016/j.eclinm.2025.103662. | Irrelevant exposure. |
| Wei Cui 2026 | 1. Cui W, Tao Y, Zhang S, Zhou W, Liu Z. Association of dietary intake patterns with treatment outcomes in late-stage pancreatic tumors. *Front Nutr*. 2026;13:1757545. Published 2026 Apr 13. doi:10.3389/fnut.2026.1757545. | Irrelevant exposure. |
| Anaïs Hasenböhler 2026 | 1. Hasenböhler A, Javaux G, Payen de la Garanderie M, et al. Intake of food additive preservatives and incidence of cancer: results from the NutriNet-Santé prospective cohort. *BMJ*. 2026;392:e084917. Published 2026 Jan 7. doi:10.1136/bmj-2025-084917. | Irrelevant exposure. |
| YaWen Tan 2026 | 1. Tan YW, Ou QJ, Xu H, et al. Associations between degree of food processing, inflammatory biomarkers and colorectal cancer survival: a prospective cohort study. *Food Funct*. Published online April 28, 2026. doi:10.1039/d6fo00196c. | Irrelevant exposure. |
| Fernanda Morales-Berstein 2024 | 1. Morales-Berstein F, Biessy C, Viallon V, et al. Ultra-processed foods, adiposity and risk of head and neck cancer and oesophageal adenocarcinoma in the European Prospective Investigation into Cancer and Nutrition study: a mediation analysis. *Eur J Nutr*. 2024;63(2):377-396. doi:10.1007/s00394-023-03270-1. | Duplicate cohort. |
| Jeroen Berden 2026 | 1. Berden J, Langselius O, González-Gil EM, et al. Assessing the role of ultra-processed foods in colorectal cancer incidence: insights from the EPIC cohort. *BMC Public Health*. Published online April 22, 2026. doi:10.1186/s12889-026-27225-3. | Duplicate cohort. |
| M Waluga 2018 | 1. Waluga M, Zorniak M, Fichna J, Kukla M, Hartleb M. Pharmacological and dietary factors in prevention of colorectal cancer. J Physiol Pharmacol. 2018;69(3):10.26402/jpp.2018.3.02. doi:10.26402/jpp.2018.3.02. | Review. |
| Sergio Ruiz-Saavedra 2023 | 1. Ruiz-Saavedra S, Zapico A, González S, Salazar N, de Los Reyes-Gavilán CG. Role of the intestinal microbiota and diet in the onset and progression of colorectal and breast cancers and the interconnection between both types of tumours. Microbiome Res Rep. 2023;3(1):6. Published 2023 Nov 27. doi:10.20517/mrr.2023.36. | Review. |
| Oscar M Laudanno 2023 | 1. Laudanno OM. Cambios en la microbiota por ultraprocesados: obesidad, cáncer y muerte prematura [Changes in the microbiota due to ultra-processed foods: obesity, cancer and premature death]. Medicina (B Aires). 2023;83(2):278-282. | Review. |
| Takahisa Matsuda 2025 | 1. Matsuda T, Fujimoto A, Igarashi Y. Colorectal Cancer: Epidemiology, Risk Factors, and Public Health Strategies. Digestion. 2025;106(2):91-99. doi:10.1159/000543921. | Review. |
| Alejandro Oncina-Cánovas 2026 | 1. Oncina-Cánovas A, Cabañas-Alite L, Comino I, Mustieles V. Adherence to Plant-Based Dietary Patterns and Digestive Cancers: A Scoping Review. Nutrients. 2026;18(5):756. Published 2026 Feb 26. doi:10.3390/nu18050756. | Review. |

# Supplementary Table S4. Meta-analysis results for the association between UPF subcategory intake and digestive system cancer risk in cohort studies.

| **UPF subcategory** | **No. of studies** | **HR (95% CI)** | **Tau²** | **Test for heterogeneity** | |
| --- | --- | --- | --- | --- | --- |
|  |  |  |  | **I^2^ (%)** | ***p*-value** |
| UPF meat/protein products | 3 | 1.33 (1.15-1.53) | 0.0092 | 44.3 | 0.1454 |
| UPF beverages | 3 | 1.04 (0.90-1.19) | 0.0095 | 46.8 | 0.1306 |
| UPF ready meals | 2 | 1.15 (0.97-1.38) | 0.0152 | 62.5 | 0.0692 |
| UPF grain/bread products | 3 | 1.13 (0.98-1.31) | 0.0098 | 46.5 | 0.1326 |
| UPF sweet snacks/desserts | 3 | 1.04 (0.95-1.15) | 0 | 0.0 | 0.6804 |

Abbreviations: CI, confidence interval; HR, hazard ratio; UPF, ultra-processed food.

The number of contributing studies for each UPF subcategory is shown in the “No. of studies” column. Because ultra-processed meat/protein products showed the clearest positive association among the subcategories, an exploratory leave-one-out sensitivity analysis was additionally conducted for this subcategory; the association remained statistically significant after sequential exclusion of individual effect estimates.

# Supplementary Table S5. Subgroup analyses of the association between UPF intake and digestive system cancer risk in cohort studies.

| **Outcome / Subgroup** | **No. of studies** | **HR (95% CI)** | **Tau²** | **Test for heterogeneity** | | ***P* for subgroup difference** |
| --- | --- | --- | --- | --- | --- | --- |
|  |  |  |  | **I² (%)** | ***p*-value** |  |
| **Overall digestive system cancers** | 9 | 1.12 (1.05-1.20) | 0.0039 | 37.6 | 0.1082 |  |
| **Sex** |  |  |  |  |  | 0.6465 |
| Female | 5 | 1.09 (1.01-1.18) | 0.0037 | 44.8 | 0.1235 |  |
| Male | 4 | 1.13 (0.97-1.32) | 0.0165 | 74.1 | 0.0090 |  |
| **Geographic region** |  |  |  |  |  | 0.6983 |
| Europe | 6 | 1.11 (1.04-1.18) | 0.0011 | 17.1 | 0.3032 |  |
| Non-Europe | 3 | 1.15 (0.97-1.37) | 0.0193 | 63.6 | 0.0413 |  |
| **Mean follow-up duration, years** |  |  |  |  |  | 0.3690 |
| < 10 | 4 | 1.17 (1.03-1.33) | 0.0062 | 39.0 | 0.1781 |  |
| ≥ 10 | 5 | 1.10 (1.01-1.19) | 0.0036 | 38.1 | 0.1517 |  |
| **Mean age at baseline, years** |  |  |  |  |  | 0.7658 |
| < 55 | 6 | 1.12 (1.04-1.20) | 0.0044 | 44.8 | 0.0924 |  |
| ≥ 55 | 3 | 1.16 (0.93-1.43) | 0.0168 | 43.6 | 0.1699 |  |
| **Mean body mass index, kg/m²** |  |  |  |  |  | 0.4190 |
| < 25 | 2 | 1.16 (1.04-1.30) | 0.0044 | 43.2 | 0.1717 |  |
| ≥ 25 | 7 | 1.10 (1.01-1.19) | 0.0040 | 35.7 | 0.1558 |  |
| **Mean total energy intake, kcal/day** |  |  |  |  |  | 0.0203 |
| < 2000 | 4 | 1.21 (1.10-1.33) | 0.0045 | 37.4 | 0.1722 |  |
| ≥ 2000 | 4 | 1.04 (0.95-1.13) | 0.0000 | 0.0 | 0.8402 |  |
| **Dietary assessment method** |  |  |  |  |  | 0.7809 |
| 24-h dietary record/recall-based assessment | 3 | 1.14 (1.02–1.27) | 0.0021 | 21.6 | 0.2792 |  |
| FFQ/dietary questionnaire-based assessment | 6 | 1.12 (1.02–1.22) | 0.0060 | 47.5 | 0.0758 |  |

Note: Effect estimates compare the highest versus lowest UPF consumption categories. *P* for subgroup difference was obtained from the chi-square test for subgroup differences.

# Supplementary Table S6. Subgroup analyses of the association between UPF intake and digestive system cancer risk in case-control studies.

| **Outcome / Subgroup** | **No. of studies** | **OR (95% CI)** | **Tau²** | **Test for heterogeneity** | | ***P* for subgroup difference** |
| --- | --- | --- | --- | --- | --- | --- |
|  |  |  |  | **I² (%)** | ***p*-value** |  |
| **Overall digestive system cancers** | 5 | 1.42 (1.25-1.60) | 0.0050 | 26.7 | 0.2433 |  |
| **Geographic region** |  |  |  |  |  | 0.3308 |
| Europe | 2 | 1.35 (1.18-1.54) | 0.0000 | 0.0 | 0.3746 |  |
| Non-Europe | 3 | 1.60 (1.16-2.19) | 0.0407 | 51.6 | 0.1266 |  |
| **Mean total energy intake, kcal/day** |  |  |  |  |  | 0.4022 |
| < 2000 | 3 | 1.36 (1.20-1.55) | 0.0000 | 0.0 | 0.5563 |  |
| ≥ 2000 | 2 | 1.95 (0.86-4.43) | 0.2801 | 75.1 | 0.0449 |  |
| **Source of controls** |  |  |  |  |  | 0.3279 |
| Cohort/population-based | 2 | 1.33 (1.15-1.53) | 0.0000 | 0.0 | 0.4401 |  |
| Hospital/visitor-based | 3 | 1.53 (1.20-1.93) | 0.0213 | 51.1 | 0.1294 |  |

Note: Effect estimates compare the highest versus lowest UPF consumption categories. P for subgroup difference was obtained from the chi-square test for subgroup differences.

Abbreviations: CI, confidence interval; HR, hazard ratio; OR, odds ratio; UPF, ultra-processed food.

# Supplementary Table S7. Meta-regression analyses of study-level modifiers for the association between UPF intake and digestive system cancer risk.

| **logHR** | **Coefficient** | **Standard error** | **t** | ***P* > \| t \|** | **95% CI** |
| --- | --- | --- | --- | --- | --- |
| Geographic region | -.0029829 | .0700102 | -0.04 | 0.970 | -.3042127–.2982468 |
| Follow-up group | -.2707141 | .2153859 | -1.26 | 0.336 | -1.197445–.6560165 |
| Baseline age group | .1032625 | .1505757 | 0.69 | 0.564 | -.5446126–.7511375 |
| Body Mass Index | -.2163207 | .1055805 | -2.05 | 0.177 | -.6705969–.2379555 |
| Mean total energy intake | -.0282989 | .0404934 | -0.70 | 0.557 | -.202528–.1459303 |
| Dietary assessment method | .3223854 | .2029456 | 1.59 | 0.253 | -.5508188–1.19559 |
| UPF exposure metric | -.0145824 | .0628097 | -0.23 | 0.838 | -.2848305–.2556658 |
| Age as covariate | .2154215 | .1307761 | 1.65 | 0.175 | -.1476712–.5785142 |
| Sex as covariate | -.3088913 | .1783843 | -1.73 | 0.158 | -.8041654–.1863828 |
| BMI as covariate | -.1647574 | .1066669 | -1.54 | 0.197 | - .4609122–.1313974 |
| Smoking as covariate | .060326 | .0925198 | 0.65 | 0.550 | -.19655–.317202 |
| Alcohol as covariate | .0395308 | .1418792 | 0.28 | 0.794 | - .3543891–.4334507 |
| Physical activity as covariate | -.3908559 | .2608584 | -1.50 | 0.273 | -1.513239–.7315273 |
| Total energy as covariate | .1517935 | .2056939 | 0.74 | 0.537 | -.7332358–1.036823 |
| Diabetes as covariate | -.1107224 | .1748288 | -0.63 | 0.591 | - .86295–.6415053 |
| Family history of cancer as covariate | -.0577892 | .1925132 | -0.30 | 0.792 | -.8861067–.7705283 |
| Medication as covariate | -.0883717 | .1634078 | -0.54 | 0.643 | -.7914585–.6147152 |
| Additional dietary quality/nutrient adjustment beyond energy | -.1638512 | .2384759 | -0.69 | 0.563 | -1.18993–.862228 |
| Total number of covariates | -.012438 | .083768 | -0.15 | 0.896 | - .3728627–.3479867 |
| _Constant | .6033699 | .5017487 | 1.20 | 0.352 | -1.555481–2.76222 |

# Supplementary Table S8. Newcastle–Ottawa Scale quality assessment of cohort studies.

| Author, year | Selection | | | | Comparability | Outcome | | | Overall  quality |
| --- | --- | --- | --- | --- | --- | --- | --- | --- | --- |
|  | Representative of cohort | Selection of cohort | Exposure ascertainment | No history of disease | Comparability of cohorts | Outcome assessment | follow-up long enough(median≥1 year) | Adequacy of follow up |  |
| Thibault Fiolet 2018 | 0 | 1 | 1 | 1 | 2 | 1 | 1 | 1 | 8 |
| Lu Wang 2022a | 0 | 1 | 1 | 1 | 2 | 1 | 1 | 1 | 8 |
| Lu Wang 2022b | 0 | 1 | 1 | 1 | 2 | 1 | 1 | 1 | 8 |
| Nathalie Kliemann 2023 | 1 | 1 | 1 | 1 | 2 | 1 | 1 | 1 | 9 |
| Kiara Chang 2023 | 0 | 1 | 1 | 1 | 2 | 1 | 1 | 1 | 8 |
| GuoChao Zhong 2023 | 0 | 1 | 1 | 1 | 2 | 1 | 1 | 1 | 8 |
| Longgang Zhao 2024a | 1 | 1 | 1 | 1 | 2 | 1 | 1 | 1 | 9 |
| Longgang Zhao 2024b | 0 | 1 | 1 | 1 | 2 | 1 | 1 | 1 | 8 |
| Rie Mols 2025 | 1 | 1 | 1 | 1 | 2 | 1 | 1 | 1 | 9 |
| Aline Al Nahas 2025 | 1 | 1 | 1 | 1 | 2 | 1 | 1 | 1 | 9 |

Wells GA, Shea D, O'Connell D, et al. The newcastle-ottawa scale (NOS) for assessing the quality of nonrandomised studies in meta-analyses. <https://www.ohri.ca/programs/clinical_epidemiology/oxford.asp>

**Selection**

1: Representativeness of the exposed cohort (1 point); 2: Selection of the non exposed cohort (1 point); 3: Ascertainment of exposure (1 point); 4: Demonstration that outcome of interest was not present at start of study (1 point).

**Comparability**

1: Comparability of cohorts on the basis of the design or analysis (2 point).

**Outcome**

1: Assessment of outcome (1 point); 2: Was follow-up long enough for outcomes to occur (1 point); 3: Adequacy of follow up of cohorts (1 point).

# Supplementary Table S9. Newcastle–Ottawa Scale quality assessment of case-control studies.

| Author, year | Selection | | | | Comparability | Exposure | | | Overall  quality |
| --- | --- | --- | --- | --- | --- | --- | --- | --- | --- |
|  | adequate case definition | Representative case | Selection of Controls | Definition of Controls | Comparability of cases | Ascertainment of exposure | Same method of ascertainment | Non-response rate |  |
| Dora Romaguera 2021 | 1 | 1 | 1 | 1 | 2 | 1 | 1 | 0 | 8 |
| Khaoula El Kinany 2022 | 1 | 1 | 0 | 1 | 2 | 1 | 1 | 0 | 7 |
| Fatemeh Jafari 2023 | 1 | 0 | 0 | 1 | 2 | 1 | 1 | 1 | 7 |
| Mengxi Du 2025 | 1 | 1 | 1 | 1 | 2 | 1 | 1 | 1 | 9 |
| Laura Torres-Collado 2026 | 1 | 1 | 0 | 1 | 2 | 1 | 1 | 1 | 8 |

Wells GA, Shea D, O'Connell D, et al. The newcastle-ottawa scale (NOS) for assessing the quality of nonrandomised studies in meta-analyses. <https://www.ohri.ca/programs/clinical_epidemiology/oxford.asp>

**Selection**

1: Is the case definition adequate (1 point); 2: Representativeness of the cases (1 point); 3: Selection of Controls (1 point); 4: Definition of Controls (1 point).

**Comparability**

1: Comparability of cases and controls on the basis of the design or analysis (2 point).

**Exposure**

1: Ascertainment of exposure (1 point); 2: Same method of ascertainment for cases and controls (1 point); 3: Non-Response rate (1 point).

# Supplementary Table S10. GRADE certainty-of-evidence assessment for the association between UPF intake and digestive system cancer risk.

| **Outcomes** | **Relative effect difference (95 % CI)** | **Absolute effect difference (95 % CI)** | **No of Participants**  **(studies)** | **Quality of the evidence**  **(GRADE)** |
| --- | --- | --- | --- | --- |
| **Study design: Cohort studies** |  |  |  |  |
| Overall digestive system cancers | HR = 1.12, 95% CI | – | 1,831,249 | ⨁⨁⨁◯ |
|  | (1.05–1.20) |  | (9) | Moderate (c+) |
| Colorectal cancer | HR = 1.14, 95% CI | – | 1,035,865 | ⨁⨁◯◯ |
|  | (1.06–1.22) |  | (5) | Low |
| Colon cancer | HR = 1.15, 95% CI | – | 724,637 | ⨁⨁◯◯ |
|  | (1.05–1.26) |  | (3) | Low |
| Proximal colon cancer | HR = 1.22, 95% CI | – | 724,637 | ⨁◯◯◯ |
|  | (1.03–1.46) |  | (3) | Very low (b-, d-) |
| Distal colon cancer | HR = 1.22, 95% CI | – | 724,637 | ⨁◯◯◯ |
|  | (1.01–1.48) |  | (3) | Very low (d-) |
| Rectal cancer | HR = 1.01, 95% CI | – | 930,885 | ⨁◯◯◯ |
|  | (0.91–1.13) |  | (4) | Very low (d-) |
| Oesophageal adenocarcinoma | HR = 1.36, 95% CI | – | 647,537 | ⨁◯◯◯ |
|  | (0.87–2.13) |  | (2) | Very low (d-) |
| Oesophageal squamous cell carcinoma | HR = 0.82, 95% CI | – | 647,537 | ⨁◯◯◯ |
|  | (0.51–1.31) |  | (2) | Very low (d-) |
| Gastric cardia cancer | HR = 0.91, 95% CI | – | 647,537 | ⨁◯◯◯ |
|  | (0.62–1.35) |  | (2) | Very low (d-) |
| Non-cardia gastric cancer | HR = 1.43, 95% CI | – | 647,537 | ⨁◯◯◯ |
|  | (1.02–2.00) |  | (2) | Very low (d-) |
| Liver cancer | HR = 0.96, 95% CI | – | 444,434 | ⨁◯◯◯ |
|  | (0.78–1.18) |  | (3) | Very low (d-) |
| Hepatocellular carcinoma | HR = 1.05, 95% CI | – | 647,537 | ⨁◯◯◯ |
|  | (0.71–1.57) |  | (2) | Very low (d-) |
| Pancreatic cancer | HR = 1.11, 95% CI | – | 745,802 | ⨁◯◯◯ |
|  | (0.84–1.45) |  | (3) | Very low (b-, d-) |
| **Study design: Case-control studies** |  |  |  |  |
| Overall digestive system cancers | OR = 1.42, 95% CI | – | 10,950 | ⨁⨁◯◯ |
|  | (1.25–1.60) |  | (5) | Low |
| Colorectal cancer | OR = 1.42, 95% CI | – | 9,732 | ⨁⨁◯◯ |
|  | (1.21–1.66) |  | (4) | Low |
| Colon cancer | OR = 1.30, 95% CI | – | 8,147 | ⨁⨁◯◯ |
|  | (1.14–1.49) |  | (2) | Low |
| Rectal cancer | OR = 1.43, 95% CI | – | 8,147 | ⨁⨁◯◯ |
|  | (1.23–1.65) |  | (2) | Low |
| **UPF subcategory** |  |  |  |  |
| UPF meat/protein products | HR = 1.33, 95% CI | – | 377,632 | ⨁⨁◯◯ |
|  | (1.15–1.53) |  | (3) | Low |
| UPF beverages | HR = 1.04, 95% CI | – | 377,632 | ⨁◯◯◯ |
|  | (0.90–1.19) |  | (3) | Very low (d-) |
| UPF ready meals | HR = 1.15, 95% CI | – | 279,367 | ⨁◯◯◯ |
|  | (0.97–1.38) |  | (2) | Very low (b-, d-) |
| UPF grain/bread products | HR = 1.13, 95% CI | – | 377,632 | ⨁◯◯◯ |
|  | (0.98–1.31) |  | (3) | Very low (d-) |
| UPF sweet snacks/desserts | HR = 1.04, 95% CI | – | 377,632 | ⨁⨁◯◯ |
|  | (0.95–1.15) |  | (3) | Low |

Abbreviations: CI, confidence interval; HR, hazard ratio; OR, odds ratio; UPF, ultra-processed food.

**Cohort studies are usually evaluated from a Low level (⨁⨁◯◯).**

⨁⨁⨁⨁: High-quality evidence suggests that the estimated risk is very likely to be close to the true risk.

⨁⨁⨁◯: Moderate-quality evidence suggests that the estimated risk is likely to be close to the true risk.

⨁⨁◯◯: Low-quality evidence suggests that confidence in the estimated risk is limited.

⨁◯◯◯: Very low-quality evidence suggests very little confidence in the estimated risk.

**Downgrading factors (certainty of evidence)**

a-: Serious (−1) or very serious (−2) limitations in study design or execution that may substantially affect the validity of the results.

b-: Serious unexplained heterogeneity or important inconsistency in results across studies, including concerns arising from subgroup analyses (−1).

c-: Some (−1) or major (−2) concerns regarding the directness of the evidence in relation to the population, intervention, comparator, or outcomes of interest.

d-: Imprecise or sparse data (−1), defined as wide confidence intervals compatible with both clinically important benefit and harm, or a small number of events and participants with limited statistical information.

e-: High likelihood of reporting bias or publication bias (−1), assessed by whether all prespecified and clinically relevant outcomes were fully reported and whether selective reporting of outcomes, time points, subgroups, or analyses was suspected.

**Upgrading factors (certainty of evidence)**

a+: Consistent evidence from two or more observational studies with no plausible confounding, showing a large effect size (relative risk [RR] > 2.0 or < 0.5) (+1).

b+: Direct evidence with a very large effect size (RR > 2.0 or < 0.5), with no serious threats to validity (+2).

c+: Evidence of a dose–response relationship across exposure levels (+1).

d+: All plausible residual confounding would be expected to reduce the observed effect, yet the effect remains evident (+1).
